# Anti-EGFR Antibodies in the Management of Advanced Colorectal Cancer

**DOI:** 10.1093/oncolo/oyad262

**Published:** 2023-09-29

**Authors:** Pashtoon Murtaza Kasi, Manuel Geroy Afable, Cameron Herting, Mariusz Lukanowski, Zhaohui Jin

**Affiliations:** Department of Oncology/Hematology, Division of Internal Medicine, Weill Cornell Medicine, Meyer Cancer Center, Englander Institute of Precision Medicine, New York, NY, USA; Medical Affairs, Eli Lilly and Company, Indianapolis, IN, USA; Medical Affairs, Eli Lilly and Company, Indianapolis, IN, USA; Medical Affairs, Eli Lilly and Company, Indianapolis, IN, USA; Department of Oncology, Mayo Clinic, Rochester, MN, USA

## Abstract

Colorectal cancer is the third most common cancer worldwide, and incidence is rising in younger individuals. Anti-EGFR antibodies, including cetuximab and panitumumab, have been incorporated into standard-of-care practice for patients with advanced disease. Herein, we review the molecular characteristics of these agents and the trials that lead to their approvals. Further, we discuss clinical implications of data regarding biomarkers that dictate treatment selection, different dosing strategies, and side effect management. Finally, we look towards the future and describe contexts in which these agents are currently being investigated clinically with a focus on combinations with MAPK-targeted therapies and immunotherapy. Overall, this review provides historical context, current clinical usage, and future directions for anti-EGFR antibodies in advanced colorectal cancer.

**Implications for Practice**

Anti-EGFR therapy has remained a mainstay in the treatment of colorectal cancer, despite a continually changing diagnostic and therapeutic landscape. The two most widely utilized anti-EGFR therapies, cetuximab and panitumumab, are similar but distinct therapies with respect to molecular characteristics and clinical application. This manuscript clarifies diagnostic tests and biomarkers which guide the application of anti-EGFR therapies and selection of combinatorial targeted therapies in colorectal cancer. Strategies for side effect management, including intermittent dosing, are provided. Finally, the manuscript concludes with a discussion of active areas of investigation with anti-EGFR therapy, providing a glimpse into future applications of these molecules.

## Introduction

Colorectal cancer (CRC) is the third most common cancer worldwide.^1^ In the US, it is the second leading cause of cancer-related death, and the lifetime risk of developing CRC is about 1 in 23 (4.3%) for men and 1 in 25 (4.0%) for women.^2,3^ Approximately, 50% of patients diagnosed with CRC develop metastases, with a majority being unresectable. The 5-year relative survival rate for all stages of CRC in an age-adjusted population from 2012 to 2018 was 65.1%.^4^ There was a decrease in the number of deaths from 23.6 in 1992 to 12.8 in 2019 per 100,000 people, which is attributed to improvements in diagnosis and treatment.^4-6^ Unfortunately, there has been an increase in the incidence among younger individuals in Western countries.^7^ As most of these individuals do not meet the age criteria for screening, they are often diagnosed at an advanced or metastatic stage.

Treatment of CRC, like many other gastrointestinal cancers, is multi-modal and multi-disciplinary. For metastatic tumors, systemic chemotherapy is universally leveraged. Multi-modality curative-intent therapy can be attempted for oligometastatic disease. This can include surgery and perioperative radiotherapy in addition to systemic therapy.^8^ A 5-fluorouracil (5-FU)-based chemotherapy constitutes the backbone of systemic therapy and is frequently combined with irinotecan (FOLFIRI), oxaliplatin (FOLFOX), or both (FOLFOXIRI, FOLFIRINOX).^9^ Nearly half of the patients treated with 5-FU-based chemotherapy develop drug resistance stemming from improved DNA repair and enhanced drug metabolism.^10^

Recent advances in tumor molecular profiling have revolutionized metastatic colorectal cancer treatment. As shown in the global, phase III KEYNOTE-177 trial, patients with microsatellite instability-high/mismatch repair deficiency (MSI-H/dMMR) tumors, may benefit from single-agent pembrolizumab.^11^ PD-1 inhibitors have been approved in a tumor-agnostic manner beyond first-line for treatment of patients with MSI-H/dMMR tumors and can be used in combination with cytotoxic T-lymphocyte-associated protein 4 inhibitors (CTLA-4).^12,13^ Unfortunately, MSI-H/dMMR tumors constitute only 4% to 5% of metastatic cancers. For the remainder microsatellite stable/mismatch repair proficient (MSS/pMMR) tumors, targeted biologic therapies provide clinical benefit when combined with cytotoxic chemotherapy.^14^

The two classes of targeted therapies, most frequently utilized in patients with MSS/pMMR metastatic CRC (mCRC), are anti-EGFR (cetuximab and panitumumab) and anti-VEGF (bevacizumab) antibodies. The selection of targeted therapy depends on the *RAS* mutational status of the tumor as anti-EGFR antibodies are only approved for patients with *RAS* wild-type tumors. The addition of these targeted therapies to cytotoxic agents improve the clinical outcomes including progression-free survival (PFS) and overall survival (OS) in patients with mCRC compared to cytotoxic agents alone and represents some of the first applications of targeted therapies in oncology.^15,16^ This review summarizes the development of anti-EGFR therapies, specifically cetuximab-based regimens for the management of advanced CRC and highlights contexts in which these drugs continue to be developed.

### Establishment of Anti-EGFR Therapy as Standard of Care in CRC and Molecular Comparison of Cetuximab and Panitumumab

Cetuximab (Erbitux^®^) and panitumumab (Vectibix^®^) are the first anti-EGFR targeted therapies that were investigated for the management of CRC and are now broadly utilized. Cetuximab is a mouse/human chimeric monoclonal antibody (mAb) targeted against human EGFR and was the first anti-EGFR antibody approved against CRC in 2004. Its efficacy and safety have been established in phase II and III clinical trials, where it demonstrated clinically significant activity in improving survival outcomes when used alone or in combination with chemotherapy. It should be noted that anti-EGFR antibodies are associated with increased toxicity, and that is detailed later in the manuscript. Panitumumab received approval in 2006 to treat EGFR-expressing mCRC after failure with or following fluoropyrimidine-, oxaliplatin-, and irinotecan-based chemotherapies. Table 1 highlights clinical trials that were instrumental in the registration of these agents in CRC.

**Table 1.** Registrational clinical trials of cetuximab and panitumumab. Trials that were instrumental to the registration of cetuximab and panitumumab are highlighted, and their results are summarized.

Study and phase	NCT identifier	Design	Population (N)	Study arm	Cohorts	Outcomes	Conclusions
BOND^112^ (phase II)	N/A	Randomized, open-label trial	Treatment refractory mCRC (329)	EGFR + chemotherapy	Arm 1: Cetuximab + irinotecan (*n* = 218) Arm 2: Cetuximab monotherapy (*n* = 111)	Arms 1 vs. 2: Rate of response: 22.9 vs. 10.8% Median time to progression: 4.1 vs. 5.1 mo (*P* < .001) Median survival time: 8.6 vs. 6.9 mo (*P* = .48) Safety: Toxic effects were more frequent in arm 1, but their severity and incidence were like those expected in treatment with irinotecan alone	Confirmed monotherapy activity of cetuximab in the refractory setting as well as the ability of cetuximab to overcome resistance to irinotecan
CRYSTAL^22^ (phase III)	NCT00154102	Randomized, open-label, parallel trial	EGFR-expressing mCRC (1198)	EGFR + chemotherapy	Arm 1: Cetuximab + FOLFIRI (*n* = 599) Arm 2: FOLFIRI alone (*n* = 599)	Arms 1 vs. 2: PFS: HR = 0.85; 95% CI, 0.72-0.99; *P* = .048 There was significant interaction between arm 1 and *KRAS* mutation status of tumor response (*P* = .03) but not for PFS (*P* = .07) or OS (*P* = .44) PFS in wild-type *KRAS* tumors: HR = 0.68, 95% CI, 0.50-0.94 Safety: AEs were more frequently reported in arm 1 Skin reactions (Gr 3): 19.7 vs. 0.2% (*P* < .001) Infusion-related reactions: 2.5 vs. 0% (*P* < .001) Diarrhea: 15.7 vs. 10.5% (*P* = .008)	Addition of cetuximab to FOLFIRI reduced the risk of progression of mCRC. Benefit of cetuximab was limited to patients with *KRAS* wild-type tumors
20020408^113^ (phase III)	NCT00113763	Randomized, open-label trial	EGFR-expressing mCRC (463)	EGFR + best supportive care	Arm 1: Panitumumab plus best supportive care (*n* = 231) Arm 2: Best supportive care (*n* = 232)	Arms 1 vs. 2: Median duration of response: 17.0 vs. 0 weeks PFS in wild-type *KRAS* mCRC: HR = 0.454 (95% CI, 0.44-0.66) favoring panitumumab arm Objective response rates also favored panitumumab (10% vs. 0%), *P* < .0001 No differences in OS Dermatologic toxicities: 90% Ocular toxicities: 15% Incidence of paronychia was 25% with 2% of severe cases Arm 1 vs. 2: Gr ≥3 Acneiform dermatitis: 7% vs. 0% Dyspnea: 5 vs. 3% One patient experienced Gr 3 event of mucosal inflammation 11% of patients treated with panitumumab reported severe toxicity leading to dose interruption	Statistically significant prolongation in PFS was observed in panitumumab vs. best supportive care arms
ASPECCT^114^ (phase III)	NCT01001377	Randomized, open-label trial	Wild-type *KRAS* mCRC (1010)	EGFR	Arm 1: Panitumumab (*n* = 499) Arm 2: Cetuximab (*n* = 500) *KRAS*-mutant population (*n* = 243; 57%)	Arms 1 vs 2: OS (median [mo], [95% CI]): 10.4, (9.4, 11.6) vs. 10.0 (9.3, 11.0) HR = 0.97 PFS (median [mo], [95% CI]): 4.2 (3.2, 4.8) vs. 4.4 (3.2, 4.8) HR = 1.00 Objective response (%, 95% CI): 22.0 (18.4, 26.0) vs. 19.8 (16.3, 23.6)	Panitumumab was statistically significantly noninferior to cetuximab for OS
20100007^115^ (phase III)	NCT01412957	Randomized, open-label trial	Wild-type *KRAS* mCRC (377)	EGFR + best supportive care	Arm 1: Panitumumab + best supportive care (*n* = 189) Arm 2: Best supportive care (*n* = 188)	Arm 1: a vs. b OS (median [mo], 95% CI): 10.0 (8.7, 11.4) vs. 7.4 (5.8, 9.3) HR = 0.73; *P* = .0096 PFS (median [mo], 95% CI): 3.6 (3.4, 5.3) vs. 1.7 (1.6, 1.9) HR = 0.51; *P* < .0001 ORR (%, 95% CI): 27 (20.8, 33.9) vs. 1.6 (0.3, 4.6) Arm 2: a vs b OS (median [mo], 95% CI): 10.0 (8.7, 11.6) vs. 6.9 (5.2, 7.9) HR=0.70; *P* = .0135 PFS (median [mo], 95% CI): 5.2 (3.5, 5.3) vs. 1.7 (1.6, 2.2) HR = 0.46; *P* < .0001 ORR (%, 95% CI): 31 (23.5, 39.3) vs. 2.3 (0.5, 6.7)	Addition of panitumumab reported an improvement in OS
PRIME^21^ (phase III)	NCT00364013	Randomized, open-label trial	Wild-type *KRAS* mCRC (1183)	EGFR + chemotherapy	Arm 1: Wild-type *KRAS* population (a) Panitumumab + FOLFOX (325) (b) FOLFOX alone (331) Arm 2: Mutant *KRAS* population (a) Panitumumab + FOLFOX (221) (b) FOLFOX alone (219)	Arm 1: a vs. b PFS (median [mo], 95% CI): 10.1 (9.3, 11.4) vs. 9.2 (7.5, 9.9) HR = 0.77; *P* = .008 OS (median [mo], 95% CI): 23.9 (20.3, 27.7) vs. 19.7 (17.6, 22.7) HR = 0.881, *P* = .17 Arm 2: a vs. b PFS (median [mo], 95% CI): 7.4 (6.3, 8.0) vs. 9.0 (7.7, 9.6) HR = 1.27; *P* = .0040 OS (median [mo], 95% CI): 15.5 (13.1, 17.6) vs. 19.2 (16.5, 21.7); HR = 1.17; *P* = .14 Arm 1 vs. 2: ORR (%, 95% CI): 57 (51.5, 62.64) vs. 48 (42.0, 53.1) Safety profile of panitumumab in patients with wild-type *RAS* mCRC was similar to those with wild-type *KRAS* mCRC	Panitumumab is not effective to treat patients with *RAS*-mutant mCRC (*KRAS/NRAS*)

Cetuximab and panitumumab differ in their molecular compositions. Cetuximab is an IgG1 mAb, while panitumumab is IgG2 mAb. This influences how these antibodies interact with the immune system when bound to target cells. IgG1 antibodies are known to interact more extensively with CD16, resulting in increased antibody-dependent cellular cytotoxicity (ADCC). This may influence how these antibodies synergize with immune therapy, a topic that will be discussed in greater detail later in the manuscript.^17-19^ Detailed differences in the immune-related functions of cetuximab and panitumumab have been recently reviewed.^20^ In addition to their different antibody isotypes, they vary in the binding location on EGFR.

The approvals of cetuximab and panitumumab in CRC were initially made irrespective of tumor mutational profile. The importance of tumor mutational profile was uncovered by the PRIME trial (NCT00364013), retrospective subgroup analysis of data from CRYSTAL trial (NCT00154102), and the phase II OPUS trial (NCT00125034).^21-23^ Pooled analysis of the CRYSTAL and OPUS trials further confirmed benefit of cetuximab plus chemotherapy in the first-line for *KRAS* wild-type mCRC vs no benefit in *KRAS*-mutated CRC.^24^ These trials established *KRAS* mutational status as a predictive biomarker. In addition to *KRAS* mutational status, tumor-sidedness is associated with response to anti-EGFR therapy in CRC.^25^ The initial observations supporting this practice came from the translational work and retrospective analyses of phase III studies, such as CALGB/SWOG 80405 (NCT00265850) and FIRE-3 (NCT00433927), which explored anti-EGFR therapy plus chemotherapy vs anti-VEGF plus chemotherapy *KRAS/NRAS* wild-type mCRC tumors.^26^

Recently the first prospective, randomized, open-label clinical trial, PARADIGM (NCT02394795) comparing anti-EGFR and anti-VEGF approaches in the first-line setting demonstrated superior mOS in patients with *RAS* wild-type, left-sided mCRC and treated with panitumumab and mFOLFOX6 compared with bevacizumab and mFOLFOX6, supporting the conclusions of previous post-hoc analyses.^27^ Real-world analysis from the National Cancer Database (NCDB) has also demonstrated similar results.^28^ Altogether, the data from CALGB 80405, FIRE-3, CRYSTAL, and PARADIGM studies show that primary tumor location is a predictive biomarker. The recent biomarker analysis from PARADIGM suggests that tumor-sidedness may be a surrogate marker for tumor biology; however, patients may be negatively hyperselected for anti-EGFR therapy with ctDNA analysis.^29^ This hypothesis warrants further study. Table 2 summarizes the trials comparing anti-EGFR and anti-VEGF approaches in mCRC.

**Table 2.** Summary of completed clinical trials and retrospective analyses comparing anti-EGFR and anti-VEGF agents in mCRC. Retrospective and prospective analyses comparing the efficacy of anti-EGFR and anti-VEGF-containing regimens are highlighted and median overall survival (mOS) between the two arms is displayed.

Clinical trial	Anti-EGFR mOS (mo)	Anti-VEGF mOS (mo)
NCDB^28^	42.9	27.5
CALGB 80405^116^	29.9	29.0
PEAK^117^	34.2	24.3
FIRE-3^43^	31.0	26.0
PARADIGM^*^^27^	37.9	34.3

^*^Prospective clinical trial.

### Biomarker Testing and Initiation of Anti-EGFR Therapy

**Figure 1.**
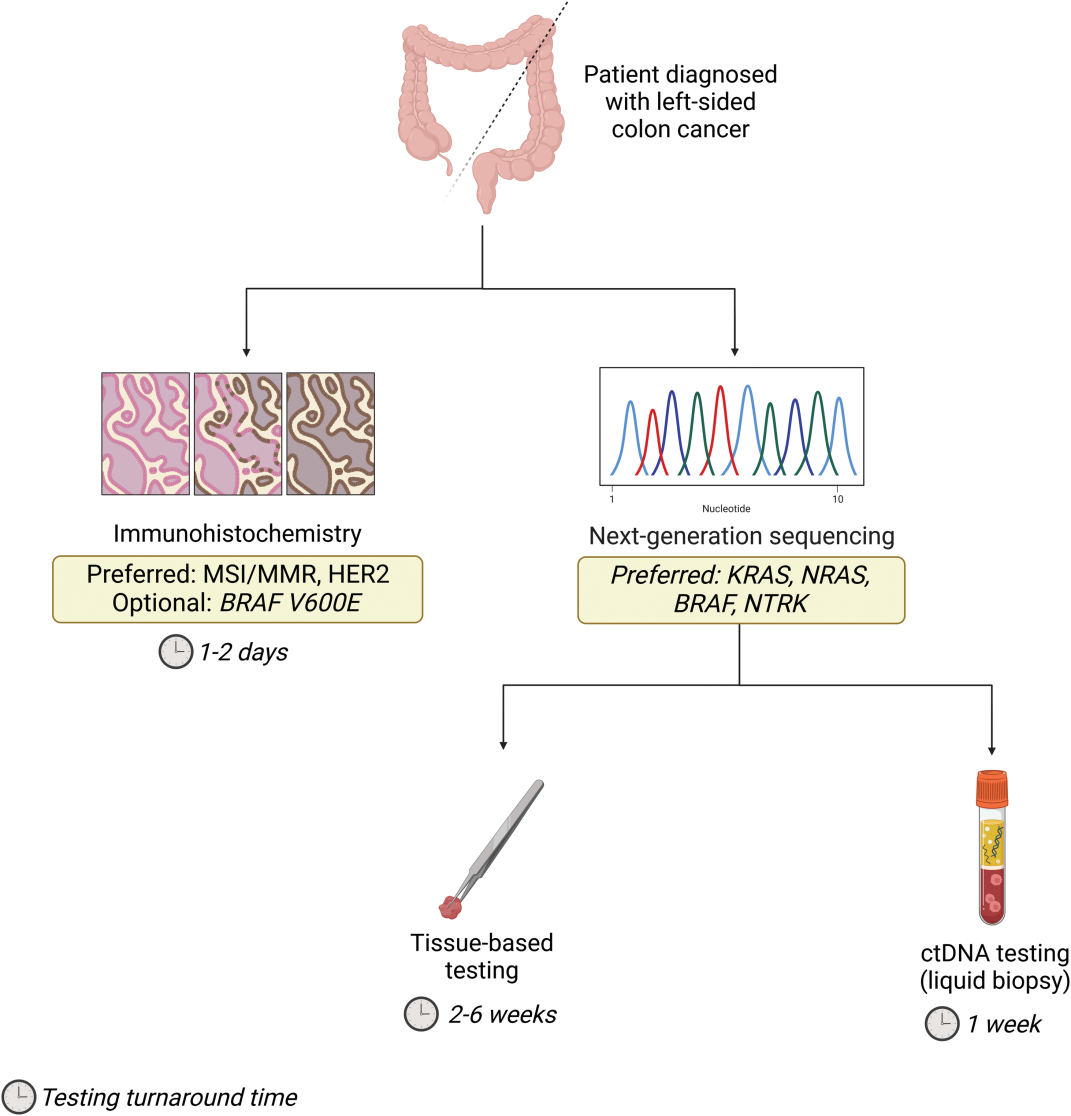
Biomarker testing flowchart. This flowchart displays biomarkers that govern selection of FDA-approved regimens in mCRC and highlights how these are typically assessed in patient samples.

As a result of these studies, *RAS* mutational status and tumor-sidedness are now frequently employed as predictive biomarkers when selecting therapy in mCRC.^30^ International treatment guidelines mandate *RAS* mutation testing prior to initiating treatment with anti-EGFR therapy.^31^ If *KRAS* mutations are detected, patients should not receive anti-EGFR therapy due to the predicted lack of benefit.^32,33^ The turnaround time for the genomic testing of tumor tissue can exceed two weeks and may delay the initiation of anti-EGFR therapy.^34^ As a result, some providers choose to initiate an anti-VEGF regimen in the first-line setting, as its use is not predicated on a particular molecular profile. Issues with managing anti-EGFR-associated toxicities like acneiform rash also cause some providers to favor first-line anti-VEGF therapy. However, with better OS outcomes from anti-EGFR therapy observed across multiple analyses, including a phase III, prospective, randomized, controlled trial, there must be an informed conversation with the patient about the choice of biologic treatment in this setting. In addition to *RAS* status, recent FDA approvals of *BRAF*^*V600E*^-targeted (BEACON) and *HER2*-targeted (MOUNTAINEER) therapies suggest that these markers should also be included in a biomarker testing schema.^35,36^  Figure 1 shows a flowchart depicting biomarker testing in left-sided mCRC.

Liquid biopsy for molecular profiling of mCRC can combat the challenges that arise from slow turnaround time of tissue-based testing.^34,37^ In this approach, tumor fragments (including cells, DNA, and methylation markers) shed into the blood circulation and can be assessed in a blood sample. It can define the risk of recurrence after curative-intent surgery and detect acquired resistance mechanisms in samples from patients exposed to targeted therapies.^34^ Liquid biopsies offer various advantages compared to tissue-based testing. These include non-invasive sample acquisition, more rapid turnaround time, detection of intratumoral and/or temporal heterogeneity, and tracking the clonal evolution of the tumor.^34^ Liquid biopsy has been endorsed as an alternative and supplemental means of genotyping to tissue-based next-generation sequencing (NGS), especially when the tissue is not available or insufficient.^38^ It should be noted, however, that liquid biopsy is limited in that it cannot assess all NCCN-recommended biomarkers in CRC. This includes HER2 which must be profiled with an immunohistochemical assay. Prospective studies such as CIRCULATE, COBRA, Dynamic II/III, and ACT3 are ongoing in the minimal residual disease (MRD) setting.^34^ In total, both tissue-based and liquid biopsies are used to assess patients for biomarkers that predict response to currently approved therapies and to determine patient eligibility for emerging therapies.

### Mechanisms of Resistance to EGFR-Targeted Therapy

Resistance to anti-EGFR therapy is common in mCRC and limits prolonged efficacy.^39^ The mechanisms of resistance include intrinsic and extrinsic tumor alterations.^40^ Various therapeutic strategies have been developed to overcome resistance through these mechanisms.^39^ An anti-EGFR therapy leads to a rise in *RAS*-mutant clones, which decline upon its withdrawal, indicating that clonal evolution continues beyond clinical progression.^41^ A complete exome sequence and copy number analysis of *KRAS* wild-type tumors showed that therapeutic resistance to anti-EGFR therapies can be overcome through combinatorial therapies targeting actionable genes.^41^ The timing of the treatment with EGFR inhibitor seemed to be crucial based on the recent adhoc analysis performed in the CALGB 80405 trial.^42^ Administration of an anti-EGFR agent in the first-line setting led to a reduction in the development of resistance mutations.

### Tumor Conversion and Anti-EGFR Rechallenge Approaches

In CRC, patients often present with oligometastatic disease with unresectable tumors at diagnosis but may be converted to resectable with treatment. In left-sided *RAS/BRAF* wild-type mCRC tumors, doublet chemotherapy plus anti-EGFR can convert tumors to resectable.^43^ Escalation of chemotherapy from a doublet to a triplet regimen was considered potentially more effective in this setting based on the phase II VOLFI trial, but the phase III TRIPLETE trial did not confirm additional benefits.^44^ Contrastingly, in first-line combinations with anti-VEGF therapy, escalation from a doublet chemotherapy (FOLFIRI) to a triplet chemotherapy (FOLFOXIRI) is associated with improved clinical outcomes albeit with increased rates of toxicity.^45^ The inclusion of cetuximab in a conversion regimen may benefit patients with symptomatic tumors.^43^

In addition to conversion studies, there is a rise in interest in rechallenging patients with EGFR-targeted therapy following progression on anti-EGFR regimens in an earlier line of therapy with an intervening non-EGFR targeted therapy.^46^ These trials are summarized in Table 3. Studies also show that the genome of patients with CRC adapts dynamically to intermittent drug schedules that provide a molecular explanation for the efficacy of anti-EGFR rechallenge therapies.^47^ Patients with *KRAS* wild-type mCRC can remain *KRAS* wild-type post-progression. These observations provide rationale for rechallenging patients with cetuximab or panitumumab. Data from these trials compare favorably with standard third-line treatments. The results from the CHRONOS trial also demonstrate that interventional liquid biopsies can guide anti-EGFR rechallenge therapy for patients with mCRC.^48^

**Table 3.** Anti-EGFR rechallenge trials. Trials investigating rechallenging patients with anti-EGFR therapy after progression in previous lines are highlighted. The table was adapted from Ciardiello D et al^46^

Study	Study type	Number of patients	Rechallenge treatment	RR (%)	mPFS (mo)	mOS (mo)
Santini et al., 2012	Retrospective	39	FOLFIRI + cetuximab Irinotecan + cetuximab	53.8	6.6	NR
CRICKET *All comers*	Prospective	28	Irinotecan + cetuximab	21.4	3.4	9.8
*RAS ctDNA wt*		13	Irinotecan + cetuximab	31	4	12.5
*RAS ctDNA mut*		12	Irinotecan + cetuximab	0	1.9	5.2
Sunakawa Y et al., 2020 *All-comers*	Prospective	16	Irinotecan + anti-EGFR	0	3.1	8.9
*RAS ctDNA wt*		10	Irinotecan + anti-EGFR	0	4.7	16
*RAS ctDNA mut*		6	Irinotecan + anti-EGFR	0	2.3	3.8
CAVE *All-comers*	Prospective	77	Cetuximab + avelumab	7.8	3.6	13.1
*RAS/BRAF/EGFR ctDNA wt*	Prospective	48	Cetuximab + avelumab	8.5	4.3	16.3
*RAS/BRAF/EGFR ctDNA mut*	Prospective	19	Cetuximab + avelumab	5.1	3	11.5
JACCRO CC-08	Prospective	34	Irinotecan + cetuximab	0	2.4	8.1
Liu et al., 2015	Prospective	89	Cetuximab ± erlotinib	NR	2.5	NR
CHRONOS	Prospective	27	Panitumumab	30	~4	12.75

Abbreviations: wt: wild-type; mut: mutant.

## Implications for Clinical Practice

### Biweekly Dosing With Cetuximab

A meta-analysis demonstrated no significant differences in efficacy and safety between biweekly and weekly cetuximab administration in patients with *KRAS* wild-type mCRC.^49^ Biweekly dosing offers multiple practical advantages, including fewer patient visits and synchronization with the biweekly schedule of chemotherapy, resulting in improved compliance and quality of life (QoL).^50^ A study comparing the cost of biweekly, first-line regimens, cetuximab and FOLFIRI vs panitumumab and FOLFOX, in US individuals with *KRAS* wild-type mCRC showed better cost savings with cetuximab and FOLFIRI among the overall population as well as different body surface areas and weights.^51^

### Cetuximab-Induced Anaphylaxis and the Alpha-Gal Test

Cetuximab is produced in the mouse cell line SP2/0 that expresses the gene for α-1,3-galactosyltransferase. This enzyme glycosylates the murine Fab region in the heavy chain of cetuximab, creating an alpha-gal (galactose-α-1,3-galactose) epitope.^49,52^ In a few people, cetuximab infusion induces hypersensitivity even at first administration, which is mediated by IgE against alpha-gal.^53^ In a retrospective analysis, the southeastern US population had a higher prevalence of hypersensitivity reactions to cetuximab.^54^ It is thought that sensitivity to alpha-gal is generated by bites from the lone star tick. Severe allergic reactions are rare but may cause death.^55,56^ Pharmacovigilance data from Korea shows that alpha-gal-specific IgE detection can accurately predict cetuximab-induced hypersensitivity before administration.^53^ As per the US Prescribing Information, the incidence of severe infusion reactions to cetuximab is 2.2%.^57^ It may be advisable to test patients for alpha-gal IgE antibodies using FDA-cleared methods before initiating cetuximab, particularly in regions, like the southeastern US, where these reactions are common. However, negative results of these tests do not completely rule out the risk of severe infusion reactions.^57^

### Toxicity, Supportive Care, and Intermittent Dosing

Treatment with anti-EGFR antibodies is also associated with skin and pulmonary toxicities as well as electrolyte imbalances.^56,57^ In the phase III non-inferiority trial (ASPECCT), adverse events (AEs) were comparable between cetuximab and panitumumab, except that the incidence of infusion reactions was lower with panitumumab.^58^ Treatment with both EGFR inhibitors was associated with incidence of the acneiform rash. Interestingly, a rash of Grade ≥2 was associated with improved survival.^59^ The phase II STEPP trial was the first study evaluating the impact of a pre-emptive skin treatment regimen on skin toxicities and QoL in patients treated with panitumumab. The incidence of specific Grade ≥2 skin toxicities during the first 6 weeks of treatment was reduced by >50% in the pre-emptive treatment group vs the reactive treatment group and was associated with improved QoL.^60^

Within days of administration, anti-EGFRs can cause electrolyte abnormalities such as hypomagnesemia, which is a common cumulative toxicity issue. Hence, patients must be monitored for electrolyte imbalances throughout their treatment and for ≥8 weeks after cessation.^57^ As shown by the IMPROVE trial and the randomized phase II trial COIN-B, intermittent dosing of cetuximab can help mitigate these toxicities, and is safe in combination with chemotherapy.^61^ Liamis G et al showed that potassium-sparing diuretics can also be of value.^62^ Guidance to manage AEs is described in the cetuximab prescribing information.^57^

## Future Development With Anti-EGFR Therapy

### Current Clinical Development of Anti-EGFR Antibodies in CRC

**Figure 2.**
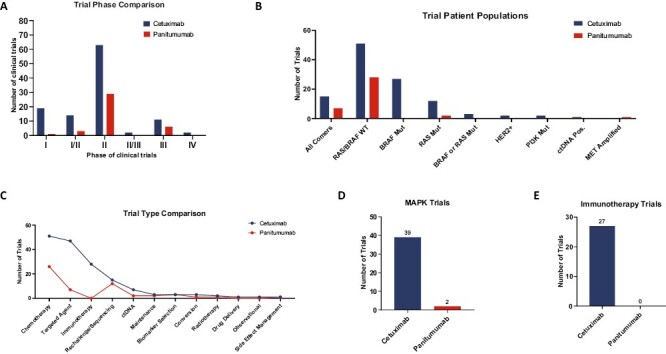
Current clinical development focus for cetuximab and panitumumab in mCRC. (**A**) The phases of open clinical trials with cetuximab and panitumumab in mCRC. (**B**) Patient populations assessed in open clinical trials with cetuximab and panitumumab in mCRC. (**C**) Illustration of drug classes paired with cetuximab and panitumumab in open trials in mCRC. Depiction of number of trials where cetuximab and panitumumab are paired with (**D**) MAPK-targeted approaches or (**E**) immunotherapy. Abbreviations: WT: wild type; mut: mutant; pos.: positive.

To comprehensively assess ongoing and planned clinical trials for cetuximab and panitumumab globally, searches were performed on Citeline/Trialtrove and clinicaltrials.gov on July 14, 2022 (Fig. 2A). The methodology used for this search is described in detail in the Supplementary Material, and Supplementary Table S1 provides a report of all included clinical trials. Both agents are primarily investigated in the *RAS/BRAF* wild-type population (Fig. 2B), a patient population for which they are currently approved in the US. Notably, cetuximab has more active trials in patients with *BRAF*-mutant and MSI-H/dMMR tumors.

We additionally assessed therapies that are being paired with cetuximab and panitumumab, and the patient population they are being studied. Both agents are frequently paired with chemotherapy in ongoing clinical trials. They are also being evaluated in rechallenge studies, sequencing studies, intermittent dosing studies, side effect management trials, and in combination with ctDNA analysis (Fig. 2C). Cetuximab has more active trials with compounds targeting the MAPK pathway (Fig. 2D) and is also the only anti-EGFR that is being evaluated in combination with immunotherapy in several trials (Fig. 2E). The immunologic differences between cetuximab and panitumumab may be influencing how these compounds are leveraged.

### Combination of Anti-EGFR Therapy With MAPK-Targeted Approaches

**Figure 3.**
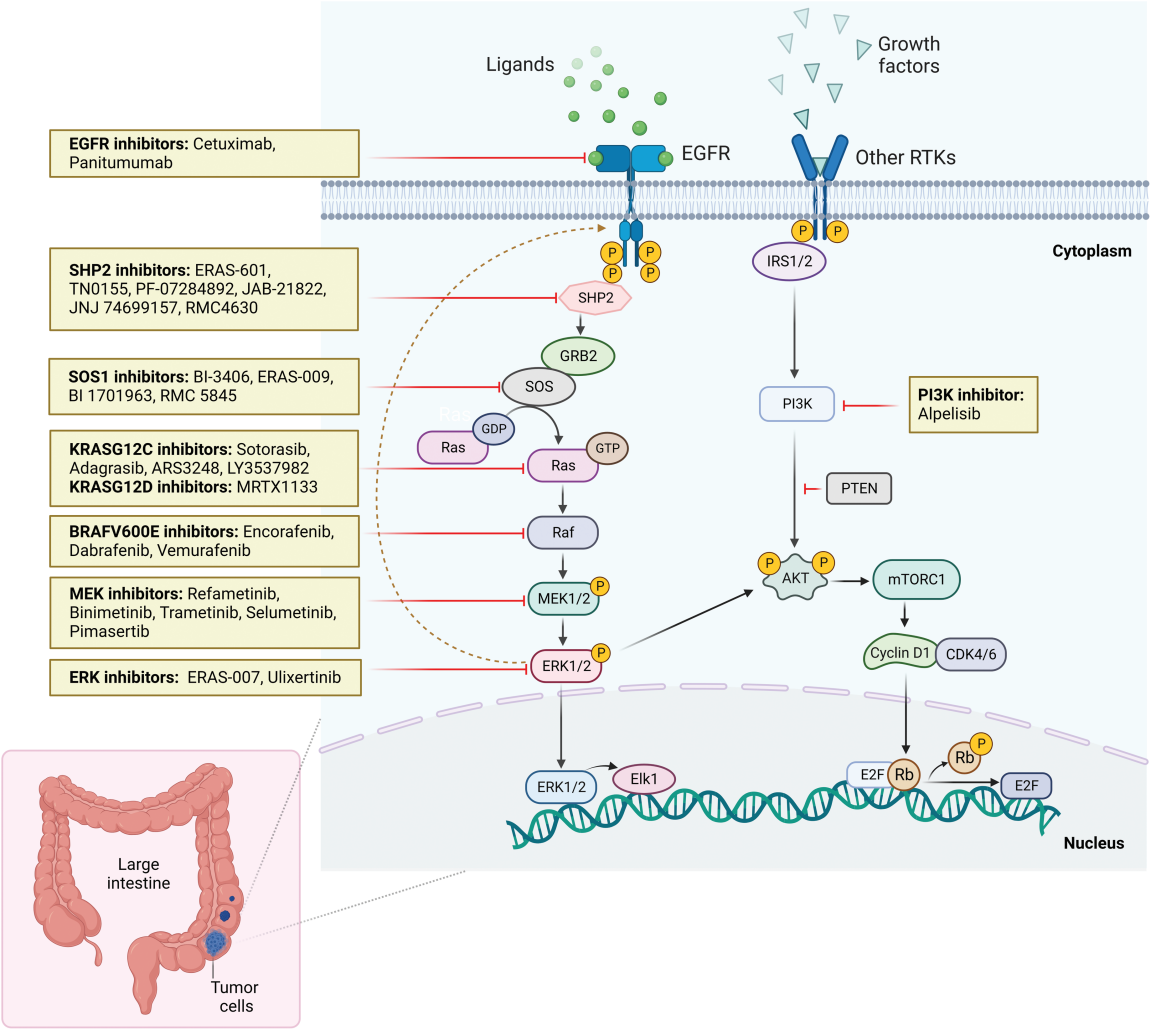
The MAPK pathway and MAPK-targeted approaches under development in CRC. The MAPK pathway is illustrated with agents that target components of the pathway depicted. The dotted arrow indicates reactivation of the MAPK pathway seen following administration of a MAPK-targeted therapy. Abbreviations: BRAF: B-RAF proto-oncogene, serine/threonine kinase; CDK4/6: cyclin-dependent kinase 4/6; EGFR: epidermal growth factor receptor; ERK1/2: extracellular signal-regulated kinase 1/2; GTP: guanosine triphosphate; MEK1/2: mitogen-activated protein kinase 1/2; mTOR: mammalian target of rapamycin; P: phosphate group; PI3K: phosphoinositide 3-kinases; PTEN: phosphatase and tensin homolog; Rb: retinoblastoma; RAF: rapidly accelerated fibrosarcoma; RAS: rat sarcoma virus; RTK: receptor tyrosine kinase; SHP2: SH2 domain-containing tyrosine phosphatase 2; SOS: son of sevenless.

The MAPK pathway was discovered over 30 years ago and plays a critical role in several physiological processes in healthy colorectal cells. Dysregulation caused by molecular aberrations in the MAPK signaling pathway results in the oncogenic activation of EGFR downstream effectors (Fig. 3), resulting in uncontrolled cell proliferation and resistance to therapy.^63,64^ As illustrated, numerous therapeutic approaches are in development that targets different components of this pathway. These are frequently paired with EGFR-targeted therapy as inhibition of MAPK can drive upstream reactivation of EGFR signaling.^64^ Targeting multiple effectors across pathways through a combination of double- or triple-targeted therapies along with chemotherapy may also help mitigate multidrug resistance. Two important and emerging therapeutic targets that are being combined with anti-EGFR therapy in CRC include BRAF and KRAS inhibitors.

### The Role of Anti-EGFR Treatment in *BRAF* ^*V600E*^-Mutated mCRC

*BRAF* mutations are found in 7% to 10% of the patients with mCRC.^65^ Of these, the most common mutation (~90%) is *BRAF*^*V600E*^. In patients with *BRAF*^*V600E*^ mCRC tumors that are treated with BRAF-inhibitor monotherapy, feedback reactivation of EGFR is observed, thereby providing rationale for combination with cetuximab or panitumumab.^66^ Data from cell lines, *in vivo* studies in *BRAF*-mutant CRC murine models,^67^ and clinical data in *BRAF*^*V600E*^ mCRC^68,69^ indicate that combined inhibition of BRAF and EGFR is synergistic. The addition of MEK inhibitors or ERK inhibitors has been proposed as additional MAPK-targeted therapies that may enhance the efficacy of combined BRAF- and EGFR-targeted therapy.^70^ Triplet therapy targeting BRAF, MEK, and EGFR has been clinically assessed in mCRC in BEACON,^71^ ANCHOR,^72^ and a phase I/II trial looking at combined dabrafenib, trametinib, and panitumumab therapy.^73,74^ In these trials, the addition of MEK inhibitors did not improve efficacy as compared with combined BRAF- and EGFR-targeted therapy. Combined cetuximab and encorafenib therapy is FDA approved for patients with previously treated, *BRAF*^*V600E*^-mutant CRC based on the results of the BEACON trial. The BREAKWATER trial is investigating the combination of “encorafenib plus cetuximab” or “encorafenib plus cetuximab plus chemotherapy” versus physician’s choice of chemotherapy as first-line therapy.^75^

An additional target within the EGFR signaling cascade that may be rationally combined with BRAF- and EGFR-targeted therapy is PI3K. The EGFR signaling activates PI3K, which activates the PI3K-AKT-mTOR pathway-involved combinations.^76^ Preclinical models have shown activation of both EGFR and PI3K signaling in *BRAF*^*V600E*^-mutant CRC cell lines.^77^ A phase Ib dose-finding study with a triple combination of encorafenib, cetuximab, and alpelisib (PI3K inhibitor) demonstrated longer PFS in *BRAF*^*V600E*^ mCRC patients with amplified EGFR.^78^ Patients with mutations in the PI3K pathway were also responsive to the addition of alpelisib. These trials indicate that combination regimens play a key role in overcoming adaptive resistance developed through CRC-specific EGFR-mediated feedback loops.^79^

### Anti-EGFR Combination Approaches in *KRAS*-Mutant CRC

A recent advent in oncology has been the development of targeted agents that specifically and potently inhibit KRAS, a target long considered undruggable.^80^ The most common *KRAS* mutation variants in human malignancies are G12X (77%) with G12D, G12V, and G12C being the most common. Similar to the experience with BRAF inhibitors, when *KRAS*^*G12C*^ mutant cell lines and tumors are treated with KRAS-inhibitor monotherapy, reactivation of upstream EGFR signaling is observed, providing rationale for combining these therapies with EGFR-targeted approaches. Preclinical studies have shown sensitivity of immortalized cell lines and *KRAS*^*G12C*^ patient-derived cells to combined cetuximab and sotorasib (KRAS inhibitor) treatment.^81^ Patient-derived xenograft models similarly exhibited tumor regression following cetuximab plus sotorasib or adagrasib administration.^81,82^ Key ongoing trials of adagrasib with cetuximab as combination partner include KRYSTAL-1 and KRYSTAL-10. Early KRYSTAL-1 trial data showed an improvement in response with adagrasib plus cetuximab (evaluable *n* = 28, ORR of 46%) vs adagrasib monotherapy (evaluable *n* = 43, ORR of 19%).^83^ The KRYSTAL-10 is a phase III trial evaluating adagrasib plus cetuximab vs chemotherapy in the second-line setting for patients with *KRAS*^*G12C*^*-*mutant advanced CRC.^84^ The CodeBreak-100 trial has also evaluated sotorasib as monotherapy and in combination with panitumumab in mCRC.^85,86^ In addition, a phase III, open-label trial CodeBreak-300 is assessing the combination of sotorasib and panitumumab vs investigator’s choice (TAS-102 or regorafenib) for previously treated, *KRAS*^*G12C*^ mutant mCRC. This work suggests that anti-EGFR antibodies may pair well with KRAS^G12C^-targeted therapies (eg, sotorasib, adagrasib, LY3537982, etc.) and KRAS^G12D^-targeted therapies (eg. MRTX1133) that are just entering early phase clinical studies.

As more patients are treated with KRAS-targeted agents in CRC, our understanding of mechanisms of resistance has progressed. There is rationale supporting the addition of SHP2, RAF, MEK, and ERK inhibitors to KRAS- and EGFR-targeted approaches. The CRISPR screens in *KRAS*^*G12D*^ preclinical models have implicated EGFR and SHP2 as possible resistance mechanisms.^87^ FLAGSHP-1 is investigating the combination of the SHP2 inhibitor ERAS-601 and cetuximab in patients with advanced solid tumors.^79,88^ Preclinical studies of the SOS1 inhibitor BI-3406 have demonstrated MAPK inhibition in pancreatic cancer and CRC cells harboring a variety of *KRAS* mutations. Combined MEK and SOS1 inhibition has also resulted in durable tumor regressions in *KRAS* mutation-driven cancer models.^89^

Preclinical studies have shown that MEK inhibitors combined with cetuximab inhibit the proliferation of CRC cells harboring various *RAS* mutations.^90,91^ Data suggest that MEK activation drives both primary and acquired resistance to cetuximab.^91^ Based on preliminary research, MEK inhibitors when combined with cetuximab or panitumumab led to regression of tumor volume in patients whose tumors were refractory to anti-EGFR therapies.^92^ A phase I trial that investigated the combination of cetuximab and the MEK1/2 inhibitor, selumetinib, demonstrated safety and tolerability with minimal antitumor activity in *KRAS*-mutant, refractory mCRC.^93^

### Combination of Anti-EGFR Therapy With Immunotherapy

**Figure 4.**
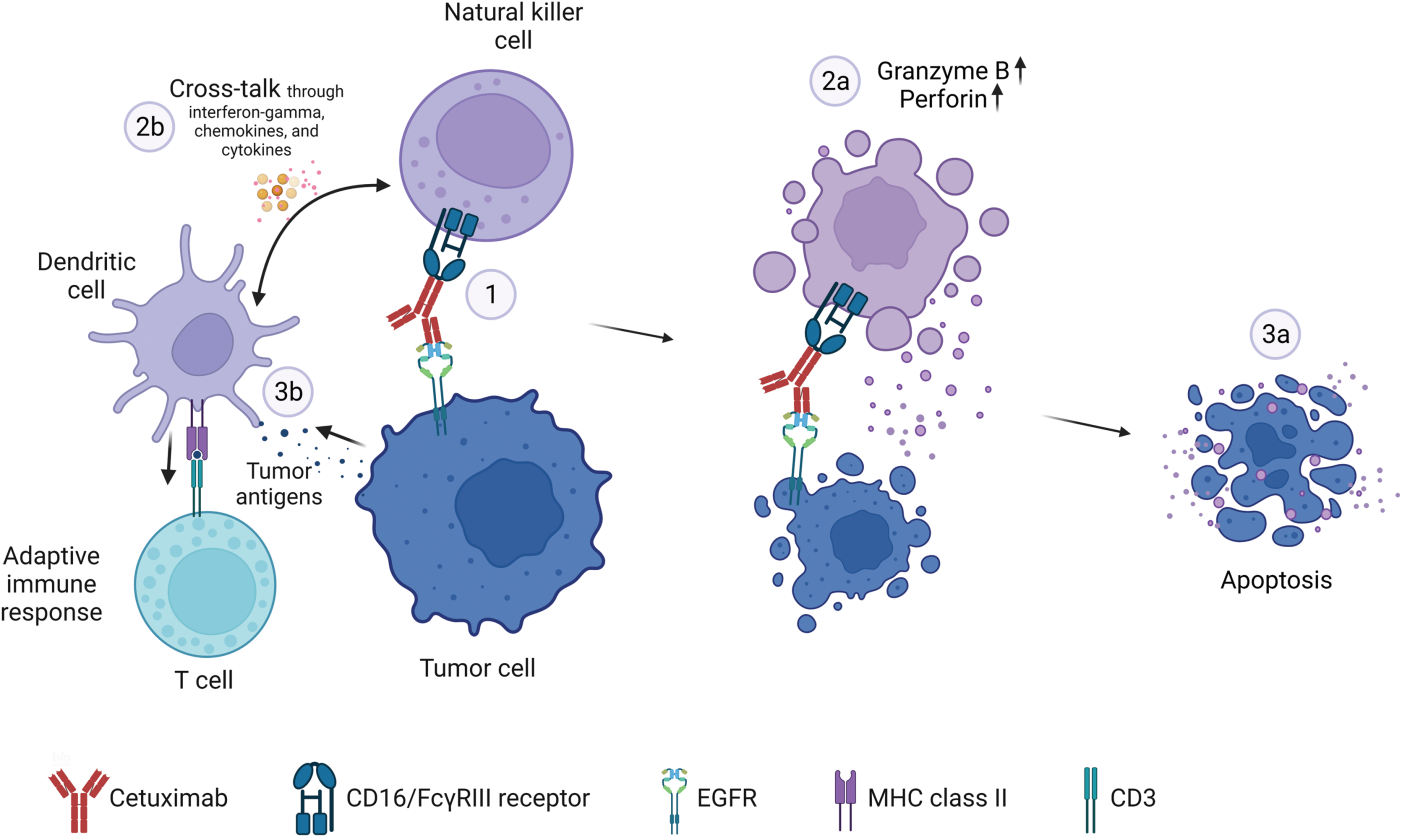
Antibody-dependent cellular cytotoxicity (ADCC). ADCC activation by cetuximab is demonstrated. The Fc region of cetuximab binds to natural killer cell receptor CD16/FcγRIII for the activation of the process 2a. Natural killer cells release tumor antigens 2b. Crosstalk between NK cells and dendritic cells through the release of interferon-gamma, chemokines, and cytokines 3a. These changes lead to tumor cell apoptosis 3b. Presentation of tumor antigens by dendritic cells to T cells leading to tumor cell lysis. Abbreviations: EGFR: epidermal growth factor receptor; Fc: fragment crystallizable.

ADCC is an immune defense mechanism that can be harnessed to target tumor cells. It is triggered by the interaction of IgG1 mAbs with natural killer (NK) cells. NK cell activation drives direct, antigen-independent, lytic activity, and the release of tumor antigens. Crosstalk between dendritic cells and NK cells occurs through the release of biochemical signals such as cytokines, chemokines, and interferon-gamma (Fig. 4).^94-98^ Tumor antigens picked up by dendritic cells are presented to T cells that can perpetuate an adaptive immune response.

Cetuximab activates ADCC through binding of its Fc region to NK cell receptor CD16/FcγRIII.^95,99^ The ability of cetuximab to stimulate ADCC can result in increased killing of EGFR^+^ CRC cells.^100^ Immune effects of cetuximab are observed regardless of tumor *RAS/RAF* mutational status in CRC.^101^ Cetuximab when combined with chemotherapy or immunotherapy has been shown to promote the recruitment of T cells, and NK cells to distal tumor sites.^94,102^ It has, therefore, been hypothesized that a combination of cetuximab and immunotherapy in CRC may elicit synergistic activity.

### Cetuximab as a Treatment Partner With PD-1/PD-L1-Targeted Agents

Immune checkpoint blockade has revolutionized treatment for patients with MSI-H/dMMR CRC.^103^ It is hypothesized that combining immune checkpoint inhibitors with cetuximab may result in improved efficacy in patients who are MSS/pMMR due to the immunostimulatory effects of cetuximab. It is thought that the anti-PD-1/PD-L1 antibodies will enhance cytotoxic lymphocyte activity while anti-EGFR antibodies induce ADCC, enhance immune cell infiltration, and induce crosstalk among immune cells. These interactions could lead to antigen-specific antitumoral cellular immunity.^17-19,104,105^ In support of this hypothesis, completed and ongoing clinical trials with combined cetuximab and avelumab therapy such as CAVE, AVETUX, AVETUXIRI, AVETRIC, and FIRE-6 have shown promising results.^106,107^ Cetuximab also has been clinically investigated with pembrolizumab. The combination was inactive in patients with *RAS* wild-type mCRC, despite altering the composition of the tumor microenvironment. The trial did not meet the coprimary endpoints of the overall response rate and 6-month PFS.^108^ Immune changes included a significant increase in intratumoral cytotoxic T cells and a trend toward increased NK cells. Finally, there is interest in combining the MAPK-targeted approaches discussed previously with both immunotherapy and cetuximab. A new trial through the SWOG (S2107) cooperative group is combining nivolumab with encorafenib and cetuximab for MSS/pMMR tumors in the second-line setting.^109^ Furthermore, the SEAMARK trial is investigating whether the addition of encorafenib and cetuximab to pembrolizumab can enhance the response seen in patients with MSI-H/dMMR CRC.^110^

### Cytokine and Cell Therapy Combinations With Cetuximab

Interleukin-15 (IL-15) is responsible for mediating the expansion, proliferation, and activation of NK cells and T cells. The IL-15 agonist NKTR-255 has been assessed in combination with cetuximab in a phase Ib/II trial in solid tumors and showed dose-dependent expansion of circulating NK and T cells. Interim results confirmed a partial response in one patient and stable disease in five patients with CRC. Emerging NK-directed cell therapies may represent additional opportunities for EGFR-mediated immune modulation. Adoptive cell therapy with NK cells in a murine CRC xenograft model with cetuximab demonstrated substantial tumor growth inhibition, primarily in EGFR-expressing tumor lines.^111^ NK-based cell therapy displays a favorable safety profile and does not depend on antigen recognition. The phase I trials of adoptive NK cell-based therapy with cetuximab are ongoing.^111^

## Conclusion

The anti-EGFR antibodies were among the first-targeted therapies to be approved in oncology. They now constitute a portion of standard of care treatment offered to patients with metastatic, left-sided, *RAS* wild-type CRC. Tissue-based testing for *RAS* status is well established as a pre-requisite for EGFR-targeted therapy in CRC, and active development aims to advance liquid biopsy due to convenience and rapid turnaround time. Despite nearly two decades since their initial approval, the anti-EGFR therapy is still actively being studied in ongoing clinical development. Current trials aim to establish optimal strategies for converting patients to resectable form of the disease as well as rechallenging with anti-EGFR therapy post-progression during earlier lines of treatment. Studies have established better dosing strategies such as biweekly dosing and intermittent administration as strategies to improve patient QoL and AE profiles. Current trials are investigating both cetuximab and panitumumab as combinatorial partners with agents targeting the MAPK pathway, including both BRAF and KRAS inhibitors. With its ability to stimulate ADCC, cetuximab is also being investigated in combination with immunotherapy. In summary, anti-EGFR therapy has revolutionized treatment for patients with mCRC, and future developments aim to impact patient care by further expanding the combination therapies.

## Supplementary Material

Supplementary material is available at *The Oncologist* online.

Acknowledgments

The authors wish to thank Maanasa Surampally, MPharm, employee of Eli Lilly India Services Private Limited, India, for providing copyediting, editorial, and production support. Illustrations were created using Biorender.com. This review was prepared according to ICMJE standards.

oyad262_suppl_Supplementary_Table_S1

oyad262_suppl_Supplementary_Material

## Data Availability

No new data were generated or analyzed in support of this research.
