# Supplementary material for: Anti-EGFR Antibodies in the Management of Advanced Colorectal Cancer
Source: Oncologist. 2023 Sep 29;28(12):1034–48. doi: 10.1093/oncolo/oyad262 (PMC11025386; doi:10.1093/oncolo/oyad262)
Supplement: oyad262_suppl_Supplementary_Table_S1 [file oyad262_suppl_supplementary_table_s1.docx]

| **Trial** | **NCT#** | **Cetuximab/Panitumumab** | **Status** | **Phase** | **Line of Therapy** | **Location** | **Pharma Sponsor/Collaborator?** | **Lilly/Amgen?** | **Sponsor** | **Population** | **Trial Type** | **Combination Partners** | **Therapeutic Target** | **IO?** | **MAPK?** | **BRAF + IO?** | **Start** | **End** |
| --- | --- | --- | --- | --- | --- | --- | --- | --- | --- | --- | --- | --- | --- | --- | --- | --- | --- | --- |
| [mXELOXIRI Combined With Molecular Targeted Drug in mCRC (TRICAP)](https://clinicaltrials.gov/ct2/show/NCT04160416) | NCT04160416 | Cetuximab | Unknown | II | 1L | China | No | No | First Affiliated Hospital of Zhejiang University | All Comers | Chemotherapy | mXELOXIRI | Antimetabolite, DNA Alkylation, Topoisomerase | No | No | No | 7-1-19 | 1-1-22 |
| [SALIRI Based Regimen as First-line Treatment for Advanced Metastatic Colorectal Cancer](https://clinicaltrials.gov/ct2/show/NCT05160896) | NCT05160896 | Cetuximab | Recruiting | II | 1L | China | No | No | Second Affiliated Hospital of Zhejiang University | All Comers | Chemotherapy | SALIRI | Thymidylate Synthase, Topoisomerase | No | No | No | 11-12-21 | 6-30-23 |
| [A Study of Cetuximab Plus Raltitrexed for Maintenance Treatment in Advanced Colorectal Cancer](https://clinicaltrials.gov/ct2/show/NCT04241731) | NCT04241731 | Cetuximab | Recruiting | II | 1L | China | No | No | Jiangsu Cancer Institute and Hospital | All Comers | Chemotherapy | Raltitrexed | Thymidylate Synthase | No | No | No | 11-28-19 | 11-28-21 |
| [LEAC-102 for Advanced Colorectal Cancer](https://clinicaltrials.gov/ct2/show/NCT02826837) | NCT02826837 | Cetuximab | Not Yet Recruiting | I/II | 1L | Taiwan | Yes | No | Taiwan Leader Biotech Corp | All Comers | Chemotherapy | LEAC-102 | Natural Product | No | No | No | 9-1-22 | 2-1-24 |
| [Utomilumab, Cetuximab, and Irinotecan Hydrochloride in Treating Patients With Metastatic Colorectal Cancer](https://clinicaltrials.gov/ct2/show/NCT03290937) | NCT03290937 | Cetuximab | Active | I | 2L | US | No | No | MD Anderson | All Comers | Chemotherapy and IO | Irinotecan, Utomilumab | Topoisomerase, 4-1BB/CD137 | Yes | No | No | 12-27-17 | 12-31-22 |
| [A Dose Escalation/Expansion Study of ERAS-601 in Patients With Advanced or Metastatic Solid Tumors (FLAGSHP-1)](https://clinicaltrials.gov/ct2/show/NCT04670679) | NCT04670679 | Cetuximab | Recruiting | I | 2L | US | Yes | No | Erasca, Inc. | All Comers | Targeted Agent | ERAS-601 | SHP2 | No | Yes | No | 12-15-20 | 5-1-24 |
| [A Study of ALX148 With Cetuximab and Pembrolizumab for Refractory Microsatellite Stable Metastatic Colorectal Cancer](https://clinicaltrials.gov/ct2/show/NCT05167409) | NCT05167409 | Cetuximab | Recruiting | II | 3L+ | US | Yes | Yes (Lilly) | Criterium, Inc. | All Comers | IO | ALX148, pembrolizumab | CD47, PD-1 | Yes | No | No | 08-01-22 | 03-01-26 |
| [ONO-7913-04: An Open-Label, Uncontrolled Study of ONO-7913, ONO-4538 and the Standard of Care FOLFOX in Combination With Bevacizumab or Cetuximab as First-Line Treatment in Patients With Unresectable Advanced or Recurrent Colorectal Cancer](https://citeline.informa.com/trials/details/407820?qId=96047d22-bf93-489d-85a2-da65a80f911f) | N/A | Cetuximab | Open | I | 1L+ | Japan | Yes | No | Ono Pharmaceutical | All Comers | IO, Chemotherapy | ONO-7913, ONO-4538, FOLFOX | CD47, PD-1, Antimetabolite, DNA Alkylation | Yes | No | No | 05-01-21 | 01-31-25 |
| [Study of NKTR 255 in Combination With Cetuximab in Solid Tumors](https://clinicaltrials.gov/ct2/show/NCT04616196) | NCT04616196 | Cetuximab | Recruiting | I/II | 3L+ | US | Yes | No | Nektar Therapeutics | All Comers | IO | NKTR-255 | IL-15 | Yes | No | No | 10-30-20 | 08-01-24 |
| [QUILT 3.071: NANT Colorectal Cancer (CRC) Vaccine](https://clinicaltrials.gov/ct2/show/NCT03563157) | NCT03563157 | Cetuximab | Active | I/II | 2L+ | US | Yes | No | ImmunityBio, Inc. | All Comers | Vaccine, Cell Therapy, Targeted Agent, Chemotherapy | NANT Vaccine, NK Cells, avelumab, ALT-803 | NANT Vaccine, NK Cells, Lymphodepletion, IL-15, PD-1 | Yes | No | No | 05-25-18 | 12-30-22 |
| [TCM Combined with Chemotherapy in Treatment of Metastatic Colorectal Cancer: A Bidirectional Cohort Study of Zhenqiliujun Anti-cancer Granules Combined with mFOLFOX6/FOLFIRI Regimen and Bevacizumab/Cetuximab in First-line Treatment of Advanced Metastatic Colorectal](https://citeline.informa.com/trials/details/401625?qId=5c31ebef-9961-40f5-ab80-e35f1883548d) | N/A | Cetuximab | Open | II | 1L | China | No | No | Hospital/Medical Center | All Comers | Targeted Agent and Chemotherapy | Zhenqiliujun Granules, FOLFOX, FOLFIRI | Natural Product, Topoisomerase, Antimetabolite, DNA Alkylation | No | No | No | 12-31-20 | 12-31-23 |
| [Targeted Polymeric Nanoparticles Loaded With Cetuximab and Decorated With Somatostatin Analogue to Colon Cancer](https://clinicaltrials.gov/ct2/show/NCT03774680) | NCT03774680 | Cetuximab | Unknown | I | Unclear | Saudi Arabia | No | No | Ahmed A. H. Abdellatif | All Comers | Drug Delivery | Cetuximab Nanoparticles | EGFR | No | No | No | 02-01-20 | 01-11-21 |
| [The Efficacy and Safety of Perioperative Chemotherapy for Locally Advanced Colorectal Cancer. - The Phase II Study](https://citeline.informa.com/trials/details/288595?qId=a5d4f1a1-4de6-457a-bbe1-708b793c4ad2) | N/A | Cetuximab | Open | II | Adjuvant/Neoadjuvant | Japan | No | No | Hospital/Medical Center | All Comers | Chemotherapy | FOLFOX, FOLFOXIRI | Topoisomerase, Antimetabolite, DNA Alkylation | No | No | No | 09-26-16 | 12-31-25 |
| [Skin Rash Study Before Chemotherapy in Colorectal & Head and Neck Cancer Patients](https://clinicaltrials.gov/ct2/show/NCT01874860) | NCT01874860 | Cetuximab | Recruiting | II | 1L+ | US | No | No | Univeristy of Louisville | All Comers | Side Effect Management and Topical Agents | Doxycycline, Hydrocortisone, Sunscreen, Moisturizer, Clindamycin, Steroid | Skin (Variable) | No | No | No | 08-01-13 | 08-01-22 |
| [Cetuximab Maintenance Treatment Versus Continuation After Induction Therapy in mCRC](https://clinicaltrials.gov/ct2/show/NCT02942706) | NCT02942706 | Cetuximab | Not Yet Recruiting | II/III | 1L | China | No | No | Ruijin Hospital | RAS/BRAF WT | Maintenance and Chemotherapy | FOLFOX, FOLFIRI | Topoisomerase, Antimetabolite, DNA Alkylation | No | No | No | 11-1-21 | 10-1-22 |
| [CAPRI 2 GOIM Study: Investigate the Efficacy and Safety of a Bio-marker Driven Cetuximab-based Treatment Regimen](https://clinicaltrials.gov/ct2/show/NCT05312398) | NCT05312398 | Cetuximab | Recruiting | II | 1L+ | Italy | No | No | University of Campania | RAS/BRAF WT | Rechallenge and Chemotherapy | FOLFIRI, FOLFOX, Irinotecan | Topoisomerase, Antimetabolite, DNA Alkylation | No | No | No | 7-15-21 | 6-15-26 |
| [Safety of Cetuximab and Trifluridin Tipiracil as the Third-line Therapy in the RASwt mCRC](https://clinicaltrials.gov/ct2/show/NCT05155124) | NCT05155124 | Cetuximab | Recruiting | I | 3L | China | No | No | Wuhan Union Hospital | RAS/BRAF WT | Chemotherapy | Trifluridin Tipiracil | DNA Synthesis | No | No | No | 12-1-21 | 4-1-23 |
| [The Efficacy and Safety of Modified XELOX(mXELOX) Plus Cetuximab vs FOLFOX Plus Cetuximab in RAS and BRAF WT mCRC Pts](https://clinicaltrials.gov/ct2/show/NCT05074966) | NCT05074966 | Cetuximab | Recruiting | III | 1L | China | No | No | Chinese Academy of Medical Sciences | RAS/BRAF WT | Chemotherapy | mXELOX | Antimetabolite, DNA Alkylation | No | No | No | 9-6-21 | 6-24-25 |
| [First-line mCapOX+Cetuximab vs. mFOLFOX6+Cetuximab for Metastatic Left-sided CRC With Wild-type RAS/BRAF Genes (CAPCET)](https://clinicaltrials.gov/ct2/show/NCT05022030) | NCT05022030 | Cetuximab | Recruiting | II | 1L | China | No | No | West China Hospital | RAS/BRAF WT | Chemotherapy | mXELOX | Antimetabolite, DNA Alkylation | No | No | No | 7-21-21 | 6-30-24 |
| [c-CetuIRI Versus Ersecond-line Irinotecan s-IRI-CetuIRI](https://clinicaltrials.gov/ct2/show/NCT04833036) | NCT04833036 | Cetuximab | Recruiting | II | 2L | China | No | No | Fudan University | RAS/BRAF WT | Chemotherapy | Irinotecan | Topoisomerase | No | No | No | 10-1-19 | 11-1-22 |
| [LY3214996 and Cetuximab Alone or in Combination With Abemaciclib for the Treatment of Unresectable or Metastatic Colorectal Cancer](https://clinicaltrials.gov/ct2/show/NCT04616183) | NCT04616183 | Cetuximab | Recruiting | I/II | 2L | US | No | No | MD Anderson | RAS/BRAF WT | Targeted Agent | Abemaciclib, LY3214996 | CDK4/6, ERK1/2 | No | Yes | No | 12-2-20 | 12-31-22 |
| [A Phase I/IIA Clinical Study Of Cetuximab Combined With Fruquintinib In The Third-Line Treatment Of RAS/BRAF Wild-Type Colorectal Cancer](https://citeline.informa.com/trials/details/384812?qId=fe68310d-51f7-49a2-beb4-f395a14d9646) | N/A | Cetuximab | Open | I/II | 3L | China | No | No | Hospital/Medical Center | RAS/BRAF WT | Targeted Agent | Fruquintinib | VEGFR1,2,3 | No | No | No | 10-1-20 | 5-1-22 |
| [A Multicentre Randomised Phase II Study of mFOLFOXIRI Versus mFOLFOX6 in Combination With Bevacizumab Or Cetuximab As First-line Treatment For Patients With Metastatic Colorectal Cancer](https://citeline.informa.com/trials/details/384794?qId=fe68310d-51f7-49a2-beb4-f395a14d9646) | N/A | Cetuximab | Open | II | 1L | China | No | No | Hospital/Medical Center | RAS/BRAF WT | Chemotherapy | mFOLFOXIRI | Topoisomerase, Antimetabolite, DNA Alkylation | No | No | No | 9-15-20 | 5-20-24 |
| [Cetuximab Plus Capecitabine as Maintenance Treatment in RAS and BRAF wt Metastatic Colorectal Cancer (CLASSIC)](https://clinicaltrials.gov/ct2/show/NCT04262635) | NCT04262635 | Cetuximab | Not Yet Recruiting | III | 1L | China | No | No | Sun Yat-Sen University | RAS/BRAF WT | Maintenance and Chemotherapy | Capecitabine | Antimetabolite | No | No | No | 9-1-20 | 3-30-24 |
| [An Exploratory Study for the Efficacy and Safety of Cetuximab in Combination with Irinotecan Re-challenge Versus Regorafenib in the Third-Line Treatment of RAS Wild-Type Metastatic Colorectal Cancer](https://citeline.informa.com/trials/details/360233?qId=fe68310d-51f7-49a2-beb4-f395a14d9646) | N/A | Cetuximab | Planned | II | 3L | China | No | No | Hospital/Medical Center | RAS/BRAF WT | Rechallenge and Chemotherapy | Irinotecan | Topoisomerase | No | No | No | 11-1-19 | 11-1-21 |
| [Study of Folfiri/Cetuximab in FcGammaRIIIa V/V Stage IV Colorectal Cancer Patients (CIFRA)](https://clinicaltrials.gov/ct2/show/NCT03874026) | NCT03874026 | Cetuximab | Recruiting | II | 1L | Italy | No | No | NCI (Naples) | RAS/BRAF WT | Biomarker Selection and Chemotherapy | FOLFIRI | Antimetabolite, Toposiomerase | No | No | No | 9-5-19 | 3-31-23 |
| [Clinical Study for Cetuximab Combined With Capecitabine Maintenance Therapy After First-line Cetuximab Combined With FOLFIRI/mFOLFOX6 Chemotherapy for RAS Wild-type Advanced Left Colorectal Cancer](https://citeline.informa.com/trials/details/338190?qId=fe68310d-51f7-49a2-beb4-f395a14d9646) | N/A | Cetuximab | Open | II | 1L | China | No | No | Hospital/Medical Center | RAS/BRAF WT | Maintenance and Chemotherapy | Capecitabine | Antimetabolite | No | No | No | 12-1-18 | 12-1-21 |
| [Palbociclib and Cetuximab in Metastatic Colorectal Cancer](https://clinicaltrials.gov/ct2/show/NCT03446157) | NCT03446157 | Cetuximab | Recruiting | II | 2L | US | Yes | Yes (Amgen) | UNC Lineberger Cancer Center | RAS/BRAF WT | Targeted Agent | Palbociclib | CDK4/6 | No | No | No | 3-13-18 | 1-13-26 |
| [Metastatic Colorectal Cancer (RAS-wildtype) After Response to First-line Treatment With FOLFIR Plus Cetuximab (AIO-KRK-0114)](https://clinicaltrials.gov/ct2/show/NCT02934529) | NCT02934529 | Cetuximab | Recruiting | III | 1L+ | Germany | No | No | University of Munich | RAS/BRAF WT | Rechallenge, Sequencing, and Chemotherapy | FOLFIRI, FOLFOX, Irinotecan | Topoisomerase, Antimetabolite, DNA Alkylation | No | No | No | 3-1-15 | 12-31-24 |
| [Cetuximab Re-challenge for Colorectal Cancer Liver Metastasis](https://clinicaltrials.gov/ct2/show/NCT04509635) | NCT04509635 | Cetuximab | Not Yet Recruiting | III | 3L | China | No | No | Fudan University | RAS/BRAF WT | Rechallenge | Unclear | Unclear | No | No | No | 9-1-20 | 8-31-24 |
| [Cetuximab as Salvage Therapy in Patients With Neo Wild-type RAS/RAF Metastatic Colorectal Cancer With Liver Metastases. (CETIDYL)](https://clinicaltrials.gov/ct2/show/NCT04189055) | NCT04189055 | Cetuximab | Recruiting | II | 2L | France | No | No | GCS IHFB Cognacq-Jay | RAS/BRAF WT | Rechallenge and ctDNA | Irinotecan | Topoisomerase | No | No | No | 1-7-20 | 7-31-23 |
| [Phase II Study of Tislelizumab Combined With Cetuximab and Irinotecan in the Treatment of Recurrent, Refractory mCRC](https://clinicaltrials.gov/ct2/show/NCT05143099) | NCT05143099 | Cetuximab | Recruiting | II | 3L+ | China | No | No | Shanghai Zhongshan Hospital | RAS/BRAF WT | IO and Chemotherapy | Tislelizumab, irinotecan | PD-1, Topoisomerase | Yes | No | No | 2-1-21 | 2-28-24 |
| [Phase II Multicenter, Randomised, Open-Label Study to Evaluate the Efficacy and Safety of Tislelizumab in combination with Cetuximab and Irinotecan versus Investigator&#39;s Choice in Patients with Previously Treated RAS Wild-type Advanced Colorectal Cancer](https://citeline.informa.com/trials/details/435018?qId=96047d22-bf93-489d-85a2-da65a80f911f) | N/A | Cetuximab | Planned | II | 3L+ | China | Yes | No | BeiGene | RAS/BRAF WT | IO and Chemotherapy | Tislelizumab, irinotecan | PD-1, Topoisomerase | Yes | No | No | 5-9-22 | 12-31-25 |
| [CAVE-2 GOIM Study: a Clinical Study of the Combination of Avelumab Plus Cetuximab as Rechallenge Strategy](https://clinicaltrials.gov/ct2/show/NCT05291156) | NCT05291156 | Cetuximab | Not Yet Recruiting | II | 3L+ | Italy | No | No | University of Campania | RAS/BRAF WT | IO and Rechallenge | Avelumab | PD-L1 | Yes | No | No | 7-1-22 | 7-1-25 |
| [A Phase II Study to Evaluate the Combination of Envafolimab and Cetuximab in the Treatment of Patients with Metastatic Colorectal Cancer (mCRC)](https://citeline.informa.com/trials/details/427600?qId=96047d22-bf93-489d-85a2-da65a80f911f) | N/A | Cetuximab | Planned | II | 2L+ | China | Yes | No | 3D Medicines | RAS/BRAF WT | IO | Envafolimab | PD-L1 | Yes | No | No | N/A | N/A |
| [Tislelizumab Plus Cetuximab and Irinotecan vs Third-line Standard-of-care in Refractory mCRC](https://clinicaltrials.gov/ct2/show/NCT05278351) | NCT05278351 | Cetuximab | Not Yet Recruiting | II | 3L+ | China | No | No | Shanghai Zhongshan Hospital | RAS/BRAF WT | IO and Chemotherapy | Tislelizumab, irinotecan | PD-1, Topoisomerase | Yes | No | No | 6-1-22 | 12-1-25 |
| [Phase II Study of Tislelizumab Combined With Cetuximab and Irinotecan in the Treatment of Recurrent, Refractory mCRC](https://clinicaltrials.gov/ct2/show/NCT05143099) | NCT05143099 | Cetuximab | Recruiting | II | 3L+ | China | No | No | Shanghai Zhongshan Hospital | RAS/BRAF WT | IO and Chemotherapy | Tislelizumab, irinotecan | PD-1, Topoisomerase | Yes | No | No | 2-1-21 | 2-28-24 |
| [SHR1316 Combined with Cetuximab and Chemotherapy in the First-Line Treatment of RAS/BRAF Wild Type Colorectal Cancer](https://citeline.informa.com/trials/details/386711?qId=96047d22-bf93-489d-85a2-da65a80f911f) | N/A | Cetuximab | Open | II | 1L | China | No | No | Hospital/Medical Center | RAS/BRAF WT | IO and Chemotherapy | SHR1316, unclear | PD-L1, unclear | Yes | No | No | 9-30-22 | 6-30-23 |
| [Clinical Trial of Camrelizumab Combined with Cetuximab and Chemotherapy Drugs to treat Ras Wild Colorectal Cancer](https://citeline.informa.com/trials/details/361769?qId=96047d22-bf93-489d-85a2-da65a80f911f) | N/A | Cetuximab | Planned | II | 2L+ | China | No | No | Hospital/Medical Center | RAS/BRAF WT | IO and Chemotherapy | Camrelizumab, unclear | PD-1, unclear | Yes | No | No | 12-1-19 | 9-30-22 |
| [AVELUMAB and CETUXIMAB and mFOLFOXIRI as Initial Therapy for Unresectable Metastatic Colorectal Cancer Patients (AVETRIC)](https://www.clinicaltrials.gov/ct2/show/NCT04513951) | NCT04513951 | Cetuximab | Active | II | 1L | Italy | No | No | Gruppo Oncologico del Nord-Ovest | RAS/BRAF WT | IO and Chemotherapy | Avelumab, FOLFOXIRI | PD-L1, Topoisomerase, Antimetabolite, DNA Alkylation | Yes | No | No | 4-1-20 | 12-31-23 |
| [Avelumab and Cetuximab in Combination With FOLFOX in Patients With Previously Untreated Metastatic Colorectal Cancer - The Phase II AVETUX-CRC Trial. (AVETUX)](https://clinicaltrials.gov/ct2/show/NCT03174405) | NCT03174405 | Cetuximab | Active | II | 1L | Germany | No | No | AIO-Studien-gGmbH | RAS/BRAF WT | IO and Chemotherapy | Avelumab, FOLFOX | PD-L1, Antimetabolite, DNA Alkylation | Yes | No | No | 7-17-17 | 7-1-22 |
| [Exploratory Study on Combined Conversion Immunotherapy for Liver Metastasis of MSS Type Initial Unresectable Colorectal Cancer Based on Gene Status](https://clinicaltrials.gov/ct2/show/NCT05409417) | NCT03174405 | Cetuximab | Recruiting | II/III | 1L+ | China | No | No | Second Affiliated Hospital of Zhejiang University | RAS/BRAF WT | IO and Chemotherapy | Tislelizumab, XELOX | PD-1 ,Antimetabolite, DNA Alkylation | Yes | No | No | 10-1-22 | 9-1-24 |
| [FOLFIRI + Cetuximab + Avelumab RAS Wild-type CRC](https://clinicaltrials.gov/ct2/show/NCT05217069) | NCT05217069 | Cetuximab | Active | II | 1L | Germany | Yes | No | University of Munich | RAS/BRAF WT | IO and Chemotherapy | Avelumab, FOLFIRI | PD-L1, Antimetabolite, Topoisomerase | Yes | No | No | 9-27-19 | 8-1-24 |
| [FT536 Monotherapy and in Combination With Monoclonal Antibodies in Advanced Solid Tumors](https://clinicaltrials.gov/ct2/show/NCT05395052) | NCT05395052 | Cetuximab | Recruiting | I | 2L+ | US | Yes | No | Fate Therapeutics | RAS/BRAF WT | Cell Therapy and Chemotherapy | FT536, Fludarabine, Cyclophosphamide, IL-2 | NK Cell, Lymphodepletion, IL-2 | Yes | No | No | 05-31-22 | 04-01-27 |
| [Monitoring RAS Phenotypic Changes Based on ctDNA to Predict the Efficacy of Cetuximab in Metastatic Colorectal Cancer](https://citeline.informa.com/trials/details/360163?qId=fe68310d-51f7-49a2-beb4-f395a14d9646) | N/A | Cetuximab | Planned | I | 1L | China | No | No | Hospital/Medical Center | RAS/BRAF WT | ctDNA, Rechallenge, Chemotherapy | FOLFOXIRI | Topoisomerase, Antimetabolite, DNA Alkylation | No | No | No | 11-01-19 | 01-31-21 |
| [The Emergence of RAS Mutations in Metastatic Colorectal Cancer Patients Receiving Cetuximab Treatment](https://clinicaltrials.gov/ct2/show/NCT03401957) | NCT03401957 | Cetuximab | Unknown | IV | 1L | Taiwan | No | No | NHRI, Taiwan | RAS/BRAF WT | ctDNA, Chemotherapy | FOLFOX, FOLFIRI, FOLFOXIRI | Topoisomerase, Antimetabolite, DNA Alkylation | No | No | No | 01-01-18 | 01-01-22 |
| [Zuojinwan Combined With Chemotherapy and Cetuximab for Advanced Colorectal Cancer: A Randomized, Double-Blind, Placebo-Controlled Clinical Trial.](https://citeline.informa.com/trials/details/382967?qId=fe68310d-51f7-49a2-beb4-f395a14d9646) | N/A | Cetuximab | Open | II | 1L+ | China | No | No | Hospital/Medical Center | RAS/BRAF WT | Targeted Agent and Rechallenge | Zuojinwan | Natural Product | No | No | No | 10-01-20 | 09-30-22 |
| [Short Course Radiotherapy Followed Intensive Chemotherapy With Delayed Surgery for Rectal Cancer With Synchronous Distant Metastasis](https://clinicaltrials.gov/ct2/show/NCT01923987) | NCT01923987 | Cetuximab | Recruiting | II | Adjuvant/Neoadjuvant | Korea | No | No | Korea Cancer Center Hospital | RAS/BRAF WT | Chemotherapy | FOLFOX, FOLFIRI | Topoisomerase, Antimetabolite, DNA Alkylation | No | No | No | 08-01-12 | 12-31-22 |
| [Conversion Therapy of RAS/BRAF Wild-Type Colorectal Cancer Patients With Initially Unresectable Liver Metastases](https://clinicaltrials.gov/ct2/show/NCT04687631) | NCT04687631 | Cetuximab | Recruiting | III | 1L | China | No | No | Fudan University | RAS/BRAF WT and Liver Met Positive | Conversion (Resectability) | FOLFOXIRI | Topoisomerase, Antimetabolite, DNA Alkylation | No | No | No | 1-14-21 | 12-31-26 |
| [Cetuximab Plus FOLFOXIRI vs Cetuximab Plus FOLFOX For CRCLM](https://clinicaltrials.gov/ct2/show/NCT03493048) | NCT03493048 | Cetuximab | Recruiting | II | 1L | China | No | No | Sun Yat-Sen University | RAS/BRAF WT and Liver Met Positive | Conversion (Resectability) | FOLFOXIRI, FOLFOX | Topoisomerase, Antimetabolite, DNA Alkylation | No | No | No | 1-1-18 | 12-30-22 |
| [A Study of ERAS-007 in Patients With Advanced Gastrointestinal Malignancies (HERKULES-3)](https://clinicaltrials.gov/ct2/show/NCT05039177) | NCT05039177 | Cetuximab | Recruiting | I/II | 1L | US | Yes | No | Erasca, Inc. | BRAF Mut | Targeted Agent | ERAS-007, encorafenib | ERK1/2, BRAF V600E | No | Yes | No | 9-20-21 | 12-31-24 |
| [A Phase II Study of Encorafenib + Binimetinib + Cetuximab in Patients with BRAF V600E-mutant Metastatic Colorectal Cancer After Refractory to Encorafenib + Cetuximab (BAYONET)](https://citeline.informa.com/trials/details/425211?qId=5c31ebef-9961-40f5-ab80-e35f1883548d) | N/A | Cetuximab | Open | II | 2L | Japan | Yes | No | Ono Pharmaceutical | BRAF Mut | Targeted Agent | Encorafenib, binimetinib | BRAF V600E, MEK1/2 | No | Yes | No | 1-1-22 | 6-30-25 |
| [The Efficacy and Safety of HLX208 in Metastatic Colorectal Cancer (mCRC) With BRAF V600E Mutation](https://www.clinicaltrials.gov/ct2/show/NCT05127759) | NCT05127759 | Cetuximab | Recruiting | II | 1L | China | Yes | No | Shanghai Henlius Biotech | BRAF Mut | Targeted Agent | HLX208 | BRAFV600E | No | Yes | No | 3-23-22 | 2-28-25 |
| [A Phase II Study of Rechallenge with Encorafenib + Binimetinib + Cetuximab in Patients with BRAF V600E-mutant Metastatic Colorectal Cancer with Prior Treatment History of Combination Therapy Including Encorafenib + Cetuximab (TRIDENTE)](https://citeline.informa.com/trials/details/426286?qId=fe68310d-51f7-49a2-beb4-f395a14d9646) | N/A | Cetuximab | Open | II | 2L | Japan | Yes | No | Ono Pharmaceutical | BRAF Mut | Rechallenge and Targeted Agent | Encorafenib, binimetinib | BRAF V600E, MEK1/2 | No | Yes | No | 1-1-22 | 6-30-25 |
| [The Efficacy of HLX208 (BRAF V600E Inhibitor) With Cetuximab for Metastatic Colorectal Cancer (mCRC) With BRAF V600E Mutation After First-line Treatment](https://clinicaltrials.gov/ct2/show/study/NCT04984369) | NCT04984369 | Cetuximab | Recruiting | II | 2L | China | Yes | No | Shanghai Henlius Biotech | BRAF Mut | Targeted Agent | HLX208 | BRAF V600E | No | Yes | No | 8-11-21 | 8-15-23 |
| [A Study Evaluating the Combination of Encorafenib and Cetuximab Versus Irinotecan/Cetuximab or Infusional 5-fluorouracil (5-FU)/Folinic Acid (FA)/Irinotecan (FOLFIRI)/Cetuximab in Chinese Patients With BRAF V600E Mutant Metastatic Colorectal Cancer. (NAUTICALCRC)](https://clinicaltrials.gov/ct2/show/NCT05004350) | NCT05004350 | Cetuximab | Recruiting | II | 2L | China | Yes | No | Pierre Fabre Medicament | BRAF Mut | Targeted Agent | Encorafenib | BRAF V600E | No | Yes | No | 9-14-21 | 4-30-24 |
| [Efficacy of VIC Regimen in BRAF Mutant Metastatic Colorectal Cancer](https://clinicaltrials.gov/ct2/show/NCT04790448) | NCT04790448 | Cetuximab | Recruiting | II | 1L | China | No | No | Sun Yat-Sen University | BRAF Mut | Targeted Agent and Chemotherapy | Vemurafenib, Irinotecan | BRAF V600E, Topoisomerase | No | Yes | No | 6-27-20 | 12-31-21 |
| [BRAF Inhibitor Encorafenib And Cetuximab Real Life Investigation of Next Generation CRC Treatment (BERING CRC)](https://clinicaltrials.gov/ct2/show/study/NCT04673955) | NCT04673955 | Cetuximab | Recruiting | IV | 2L | Germany | Yes | No | Pierre Fabre Pharma GmbH | BRAF Mut | Observational | Encorafenib | BRAF V600E | No | Yes | No | 9-3-20 | 9-30-26 |
| [A Study of Encorafenib Plus Cetuximab With or Without Chemotherapy in People With Previously Untreated Metastatic Colorectal Cancer](https://clinicaltrials.gov/ct2/show/NCT04607421) | NCT04607421 | Cetuximab | Recruiting | III | 1L | International | Yes | No | Pfizer | BRAF Mut | Targeted Agent, Chemotherapy | Encorafenib, FOLFOX, FOLFIRI, FOLFOXIRI | BRAF V600E, Topoisomerase, Antimetabolite, DNA Alkylation | No | Yes | No | 12-21-20 | 11-15-26 |
| [Cetuximab and Vemurafenib Plus FOLFIRI for BRAF V600E Mutated Advanced Colorectal Cancer (IMPROVEMENT)](https://clinicaltrials.gov/ct2/show/NCT03727763) | NCT03727763 | Cetuximab | Recruiting | II | 1L | China | No | No | Shanghai Changzheng Hospital | BRAF Mut | Targeted Agent, Chemotherapy | Vemurafenib, FOLFIRI | BRAF V600E, Topoisomerase, Antimetabolite | No | Yes | No | 10-8-18 | 12-31-22 |
| [TR Study Of "A Multi-Center Proof-Of-Concept Phase II Study of Encorafenib + Binimetinib + Cetuximab In Patients With BRAF Non-V600E Mutated Metastatic Colorectal Cancer (BIGBANG Study)"](https://citeline.informa.com/trials/details/322430?qId=fe68310d-51f7-49a2-beb4-f395a14d9646) | N/A | Cetuximab | Open | II | 1L | Japan | No | No | Aichi Cancer Center | BRAF Mut | Targeted Agent | Encorafenib, binimetinib | BRAF V600E, MEK1/2 | No | Yes | No | 5-15-18 | N/A |
| [Investigating the Efficacy and Safety of Encorafenib + Binimetinib + Cetuximab Therapy for Patients with RAS Wild-type and BRAF V600E Mutation-recurrent Colorectal Cancer who Relapsed During or After Adjuvant Chemotherapy After Radical Resection of Colorectal Cancer (TRESBIEN study)](https://citeline.informa.com/trials/details/425803?qId=a5d4f1a1-4de6-457a-bbe1-708b793c4ad2) | N/A | Cetuximab | Open | II | 2L | Japan | No | No | Hospital/Medical Center | BRAF Mut | Targeted Agent | Encorafenib, binimetinib | BRAF V600E, MEK1/2 | No | Yes | No | 1-13-22 | 12-31-26 |
| [BEACON Regimen With Cetuximab Every Second Week - Cetuximab Given Every Second Week with Encorafenib in Pre-Treated Patients with BRAFV600E Mutated Metastatic Colorectal Cancer. A Nordic Phase II Study.](https://citeline.informa.com/trials/details/394531?qId=a5d4f1a1-4de6-457a-bbe1-708b793c4ad2) | N/A | Cetuximab | Open | II | 2L | Europe | No | No | Hospital/Medical Center | BRAF Mut | Targeted Agent | Encorafenib | BRAF V600E | No | Yes | No | 11-1-21 | ??? |
| [Encorafenib, Binimetinib and Cetuximab in Subjects With Previously Untreated BRAF-mutant ColoRectal Cancer (ANCHOR-CRC)](https://clinicaltrials.gov/ct2/show/NCT03693170) | NCT03693170 | Cetuximab | Active | II | 1L | International | Yes | No | Pierre Fabre Medicament | BRAF Mut | Targeted Agent | Encorafenib, binimetinib | BRAF V600E, MEK1/2 | No | Yes | No | 1-17-19 | 10-29-22 |
| [Study of Encorafenib + Cetuximab Plus or Minus Binimetinib vs. Irinotecan/Cetuximab or FOLFIRI/Cetuximab With a Safety Lead-in of Encorafenib + Binimetinib + Cetuximab in Patients With BRAF V600E-mutant Metastatic Colorectal Cancer (BEACON CRC)](https://clinicaltrials.gov/ct2/show/NCT02928224) | NCT02928224 | Cetuximab | Active | III | 2L+ | International | Yes | No | Pfizer | BRAF Mut | Targeted Agent | Encorafenib, binimetinib | BRAF V600E, MEK1/2 | No | Yes | No | 10-13-16 | 9-30-22 |
| [FOLFOXIRI Plus Cetuximab vs. FOLFOXIRI Plus Bevacizumab 1st-line in BRAF-mutated mCRC (AIO-KRK-0116)](https://clinicaltrials.gov/ct2/show/NCT04034459) | NCT04034459 | Cetuximab | Active | II | 1L | Germany | No | No | University of Munich | BRAF Mut | Chemotherapy | FOLFOXIRI | Topoisomerase, Antimetabolite, DNA Alkylation | No | No | No | 11-25-16 | 12-31-23 |
| [Vemurafenib, Cetuximab, and Irinotecan Hydrochloride in Treating Patients With Solid Tumors That Are Metastatic or That Cannot Be Removed by Surgery](https://clinicaltrials.gov/ct2/show/NCT01787500) | NCT01787500 | Cetuximab | Active | I | 2L+ | US | Yes | No | MD Anderson | BRAF Mut | Targeted Agent and Chemotherapy | Vemurafenib, Irinotecan | BRAF V600E, Topoisomerase | No | Yes | No | 2-15-13 | 3-31-24 |
| [A Study to Learn About the Study Medicine Called PF-07799933 in People With Advanced Solid Tumors.](https://clinicaltrials.gov/ct2/show/NCT05355701) | NCT05355701 | Cetuximab | Recruiting | I | 2L+ | US | Yes | No | Pfizer | BRAF Mut | Targeted Agent | PF-07799933/ARRY-440, binimetinib | BRAF V600E, MEK1/2 | No | Yes | No | 7-5-22 | 6-27-27 |
| [An Umbrella Study to Explore Precision Treatment of Colorectal Cancer Based on Molecular Typing (Phase II)](https://citeline.informa.com/trials/details/385647?qId=96047d22-bf93-489d-85a2-da65a80f911f) | N/A | Cetuximab | Planned | II | 2L+ | China | No | No | Hospital/Medical Center | BRAF Mut | Targeted Agent | Undisclosed | MEK | No | Yes | No | 9-30-20 | 5-31-23 |
| [CGX1321 in Subjects With Advanced Solid Tumors and CGX1321 With Pembrolizumab or Encorafenib + Cetuximab in Subjects With Advanced GI Tumors (Keynote 596)](https://clinicaltrials.gov/ct2/show/NCT02675946) | NCT02675946 | Cetuximab | Recruiting | I | 1L+ | US | Yes | No | Curegenix Inc. | BRAF Mut | Targeted Agent | Encorafenib, CGX1321 | BRAF V600E, Wnt | No | Yes | No | 2-1-16 | 3-1-23 |
| [Testing the Addition of Nivolumab to Standard Treatment for Patients With Metastatic or Unresectable Colorectal Cancer That Have a BRAF Mutation](https://clinicaltrials.gov/ct2/show/NCT05308446) | NCT05308446 | Cetuximab | Recruiting | II | 2L+ | US | No | No | NCI | BRAF Mut | IO and Targeted Agent | Encorafenib, nivolumab | BRAF V600E, PD-1 | Yes | Yes | Yes | 6-6-22 | 8-31-24 |
| [Neoadjuvant encorafenib, binimetinib and cetuximab for patients with BRAF V600E mutated/pMMR localized colorectal cancer](https://citeline.informa.com/trials/details/433632?qId=fe68310d-51f7-49a2-beb4-f395a14d9646) | N/A | Cetuximab | Planned | II | Adjuvant/Neoadjuvant | Europe | Yes | No | Merck KGaA | BRAF Mut | Targeted Agent | Encorafenib, binimetinib | BRAF V600E, MEK1/2 | No | Yes | No | 08-22-22 | 07-31-24 |
| [Identification and Treatment Of Micrometastatic Disease in Stage III Colon Cancer](https://clinicaltrials.gov/ct2/show/NCT03803553) | NCT03803553 | Cetuximab | Recruiting | III | 1L | US | No | No | Massachusetts General Hospital | BRAF Mut | Targeted Agent, ctDNA | Encorafenib, binimetinib | BRAF V600E, MEK1/2 | No | Yes | No | 01-20-20 | 02-01-23 |
| [Encorafenib, Cetuximab, and Nivolumab in Treating Patients With Microsatellite Stable, BRAFV600E Mutated Unresectable or Metastatic Colorectal Cancer](https://clinicaltrials.gov/ct2/show/NCT04017650) | NCT04017650 | Cetuximab | Recruiting | I/II | 2L+ | US | No | No | MD Anderson | BRAF Mut and MSI-S | IO and Targeted Agent | Encorafenib, nivolumab | BRAF V600E, PD-1 | Yes | Yes | Yes | 6-14-19 | 6-30-25 |
| [Tolerability and Safety of Vemurafenib, Cetuximab Combined With Camrelizumab for BRAF V600E-mutated /MSS Metastatic Colorectal Cancer](https://clinicaltrials.gov/ct2/show/NCT05019534) | NCT05019534 | Cetuximab | Recruiting | I | 2L+ | China | No | No | West China Hospital | BRAF Mut and MSI-S | IO and Targeted Agent | Vemurafenib, Camrelizumab | BRAF V600E, PD-1 | Yes | Yes | Yes | 8-15-21 | 12-1-22 |
| [A Study of Encorafenib Plus Cetuximab Taken Together With Pembrolizumab Compared to Pembrolizumab Alone in People With Previously Untreated Metastatic Colorectal Cancer (SEAMARK)](https://clinicaltrials.gov/ct2/show/NCT05217446) | NCT05217446 | Cetuximab | Recruiting | II | 1L | International | Yes | Yes (Lilly) | Pfizer | BRAF Mut and MSI-H/d-MMR | IO and Targeted Agent | Encorafenib, pembrolizumab | BRAF V600E, PD-1 | Yes | Yes | Yes | 7-14-22 | 3-28-27 |
| [Phase 3 Study of MRTX849 With Cetuximab vs Chemotherapy in Patients With Advanced Colorectal Cancer With KRAS G12C Mutation (KRYSTAL-10)](https://clinicaltrials.gov/ct2/show/NCT04793958) | NCT04793958 | Cetuximab | Recruiting | III | 2L | International | Yes | No | Mirati Therapeutics | RAS Mut | Targeted Agent | Adagrasib | KRAS G12C | No | Yes | No | 3-15-21 | 12-30-24 |
| [A Study of VS-6766 and Cetuximab in Patients With Advanced Colorectal Cancer](https://clinicaltrials.gov/ct2/show/NCT05200442) | NCT05200442 | Cetuximab | Not Yet Recruiting | I/II | 2L+ | US | Yes | No | University of Chicago | RAS Mut | Targeted Agent | VS-6766 | RAF/MEK | No | Yes | No | 4-1-22 | 4-1-24 |
| [Multicenter, Prospective, Single-arm Study Investigating the Efficacy and Safety of Second-line Cetuximab plus Chemotherapy Treatment in Initially RAS-mt mCRC Patients Who Converted to RAS-wt at the Time of First Progression](https://citeline.informa.com/trials/details/412439?qId=fe68310d-51f7-49a2-beb4-f395a14d9646) | N/A | Cetuximab | Planned | II | 2L | Europe | Yes | No | Merck KGaA | RAS Mut | Sequencing and Chemotherapy | N/A | N/A | No | No | No | N/A | N/A |
| [A Study of IBI351 in Combination With Cetuximab in Subjects With KRAS G12C Mutated Metastatic Colorectal Cancer](https://clinicaltrials.gov/ct2/show/NCT05497336) | NCT05497336 | Cetuximab | Not Yet Recruiting | I | 1L | China | Yes | No | Innovent Biologics | RAS Mut | Targeted Agent | IBI351 | KRAS G12C | No | Yes | No | 8-30-22 | 9-30-24 |
| [Cetuximab in Third Line for Mutant APC, TP53 and RAS Patients With Refractory Metastatic Colorectal Cancer](https://clinicaltrials.gov/ct2/show/study/NCT04853043) | NCT04853043 | Cetuximab | Recruiting | II | 3L | US | No | No | University of Utah | RAS Mut | Biomarker Selection | N/A | N/A | No | No | No | 11-3-21 | 7-1-26 |
| [JAB-21822 in Combination With Cetuximab in Patients With Advanced CRC and Other Solid Tumors With KRAS G12C Mutation](https://clinicaltrials.gov/ct2/show/NCT05194995) | NCT05194995 | Cetuximab | Recruiting | I/II | 2L+ | China | Yes | No | Jacobio Pharmaceuticals Co., Ltd. | RAS Mut | Targeted Agent | JAB-21822 | KRAS G12C | No | Yes | No | 2-17-22 | 1-1-26 |
| [A Study to Evaluate the Safety, Pharmacokinetics, and Activity of GDC-6036 Alone or in Combination in Participants With Advanced or Metastatic Solid Tumors With a KRAS G12C Mutation](https://www.clinicaltrials.gov/ct2/show/NCT04449874) | NCT04449874 | Cetuximab | Recruiting | I | 1L+ | International | Yes | No | Genentech | RAS Mut | Targeted Agent | GDC-6036 | KRAS G12C | No | Yes | No | 6-29-20 | 8-2-23 |
| [Platform Study of JDQ443 in Combinations in Patients With Advanced Solid Tumors Harboring the KRAS G12C Mutation (KontRASt-03)](https://clinicaltrials.gov/ct2/show/NCT05358249) | NCT05358249 | Cetuximab | Not Yet Recruiting | I/II | 2L+ | Belgium | Yes | No | Novartis | RAS Mut | Targeted Agent | JDQ443 | KRAS G12C | No | Yes | No | 8-29-22 | 6-27-25 |
| [Phase 1/2 Study of MRTX849 in Patients With Cancer Having a KRAS G12C Mutation KRYSTAL-1](https://clinicaltrials.gov/ct2/show/NCT03785249) | NCT03785249 | Cetuximab | Recruiting | I/II | 2L+ | US | Yes | No | Mirati Therapeutics | RAS Mut | Targeted Agent | Adagrasib | KRAS G12C | No | Yes | No | 1-15-19 | 9-1-23 |
| [MoLiMoR - A Study With FOLFIRI-based First-line Therapy With or Without Intermittent Cetuximab](https://clinicaltrials.gov/ct2/show/NCT04554836) | NCT04554836 | Cetuximab | Active | II | 1L | Germany | No | No | TheraOp | RAS Mut | Chemotherapy and ctDNA | FOLFIRI | Topoisomerase, Antimetabolite | No | No | No | 12-29-20 | 10-01-24 |
| [Study in mCRC Patients RAS/BRAF wt Tissue and RAS Mutated LIquid BIopsy to Compare FOLFIRI Plus CetuxiMAb or BevacizumaB (LIBImAb)](https://clinicaltrials.gov/ct2/show/NCT04776655) | NCT04776655 | Cetuximab | Recruiting | III | 1L | Italy | No | No | Azienda Unita Sanitaria Locale Reggio Emilia | RAS Mut | Chemotherapy | FOLFIRI | Topoisomerase, Antimetabolite | No | No | No | 04-30-21 | 04-29-24 |
| [Phase 1/2 Study to investigate the Safety, Pharmacokinetics, Pharmacodynamics and Clinical activity of the Combination of Cetuximab, Cobimetinib and Palbociclib in Subjects with K-RAS wild-type BRAF V600E mutated, or K-RAS mutated, Advanced or Metastatic Colorectal cancer](https://citeline.informa.com/trials/details/417577?qId=fe68310d-51f7-49a2-beb4-f395a14d9646) | N/A | Cetuximab | Planned | I/II | 2L+ | New Zealand/Australia | Yes | No | Cothera Bioscience | BRAF/RAS Mut | Targeted Agent | Cobimetinib, palbociclib | MEK1/2, CKD4/6 | No | Yes | No | 12-6-21 | 3-19-24 |
| [A Study of LY3214996 Administered Alone or in Combination With Other Agents in Participants With Advanced/Metastatic Cancer](https://clinicaltrials.gov/ct2/show/NCT02857270) | NCT02857270 | Cetuximab | Active | I | 2L+ | International | Yes | Yes (Lilly) | Eli Lilly and Company | BRAF/RAS Mut | Targeted Agent | LY3214996, encorafenib | ERK1/2, BRAF | No | Yes | No | 9-29-16 | 9-10-22 |
| [PF-07284892 in Participants With Advanced Solid Tumors](https://clinicaltrials.gov/ct2/show/NCT04800822) | NCT04800822 | Cetuximab | Recruiting | I | 2L+ | US | Yes | No | Pfizer | BRAF/RAS Mut | Targeted Agent | ARRY-558, encorafenib | PNTP11/BRAF | No | Yes | No | 3-17-21 | 2-4-27 |
| [Study of Neratinib +Trastuzumab or Neratinib + Cetuximab in Patients With KRAS/NRAS/BRAF/PIK3CA Wild-Type Metastatic Colorectal Cancer by HER2 Status](https://clinicaltrials.gov/ct2/show/NCT03457896) | NCT03457896 | Cetuximab | Active | II | 1L+ | US | Yes | No | NSABP Foundation Inc. | HER2+ | Targeted Agent | Neratinib | HER2 | No | No | No | 5-18-18 | 9-30-22 |
| [MEN1611 With Cetuximab in Metastatic Colorectal Cancer (C-PRECISE-01) (C-PRECISE-01)](https://clinicaltrials.gov/ct2/show/NCT04495621) | NCT04495621 | Cetuximab | Recruiting | I/II | 2L+ | International | No | No | Menarini Group | PIK3CA Mut | Targeted Agent | MEN1611 | PI3K | No | No | No | 7-20-20 | 7-1-23 |
| [A Study Evaluating the Safety and Efficacy of Targeted Therapies in Subpopulations of Patients With Metastatic Colorectal Cancer (INTRINSIC)](https://clinicaltrials.gov/ct2/show/NCT04929223) | NCT04929223 | Cetuximab | Recruiting | I | 2L+ | International | Yes | No | Hoffmann-La Roche | PIK3CA Mut or RAS Mut | Targeted Agent and Chemotherapy | Inavolisib, GDC-6036. FOLFOX | PI3K, KRAS G12C, Antimetabolite, DNA Alkylation | No | Yes | No | 10-22-21 | 12-31-24 |
| [An Umbrella Study to Explore Precision Treatment of Colorectal Cancer Based on Molecular Typing (Phase I)](https://citeline.informa.com/trials/details/361955?qId=96047d22-bf93-489d-85a2-da65a80f911f) | N/A | Cetuximab | Planned | I | 2L+ | China | No | No | Shanghai East Hospital | BRAF or HER2 Mut | Targeted Agent and Chemotherapy | Pyrotinib, Irinotecan, Undisclosed | HER2, Topoisomerase, MEK | No | Yes | No | 12-01-19 | 09-30-22 |
| [Avelumab Combined With Cetuximab and Irinotecan for Treatment Refractory Metastatic Colorectal Microsatellite Stable Cancer (AVETUXIRI)](https://www.clinicaltrials.gov/ct2/show/NCT03608046) | NCT03608046 | Cetuximab | Recruiting | II | 2L+ | Belgium | No | No | Universite Catholique de Louvain | RAS/BRAF WT and non-MSI-H/dMMR | IO and Chemotherapy | Avelumab, Irinotecan | PD-L1, Topoisomerase | Yes | No | No | 10-03-18 | 12-31-23 |
| [TTX-080 HLA-G Antagonist in Subjects With Advanced Cancers](https://clinicaltrials.gov/ct2/show/NCT04485013) | NCT04485013 | Cetuximab | Recruiting | I | 2L+ | US | Yes | No | Tizona Therapeutics | RAS/BRAF WT and non-MSI-H/dMMR | IO | TTX-080 | HLA-G | Yes | No | No | 07-14-20 | 06-01-24 |
| [Efficacy and Safety of Sintilimab Combined With Regorafenib and Cetuximab / Sintilimab Combined With Regorafenib in Posterior Line Therapy of Advanced Colorectal Cancer (Regosinti)](https://www.clinicaltrials.gov/ct2/show/NCT04745130) | NCT04745130 | Cetuximab | Not Yet Recruiting | II | 2L+ | China | No | No | Tianjin Medical Univeristy Cancer Institute and Hospital | Non-MSI-H/d-MMR | IO and Targeted Agent | Sintilimab, Regorafenib | PD-1, multikinase | Yes | No | No | 02-01-21 | 12-31-22 |
| [A Study of SAR444245 Combined With Other Anticancer Therapies for the Treatment of Participants With Gastrointestinal Cancer (Master Protocol) (Pegathor Gastrointestinal 203)](https://clinicaltrials.gov/ct2/show/NCT05104567) | NCT05104567 | Cetuximab | Recruiting | II | 1L+ | International | Yes | No | Sanofi | Non-MSI-H/d-MMR | IO | SAR444245 | IL-2 | Yes | No | No | 12-09-21 | 01-23-24 |
| [A PHASE IB STUDY OF IMMUNOTHERAPY WITH EX VIVO PRE-ACTIVATED AND EXPANDED CB-NK CELLS IN COMBINATION WITH CETUXIMAB, IN COLORECTAL CANCER PATIENTS WITH MINIMAL RESIDUAL DISEASE (MRD)](https://clinicaltrials.gov/ct2/show/NCT05040568) | NCT05040568 | Cetuximab | Recruiting | I | 2L+ | US | No | No | MD Anderson | ctDNA Positive (MRD) | Cell Therapy and Chemotherapy | NK Cells, Fludarabine, Cyclophosphamide | Expanded NK Cell, Antimetabolite, DNA Alkylation | Yes | No | No | 02-28-22 | 06-30-23 |
| [Phase II Study Comparing Conversion Rate to Surgery With Hepatic Arterial Infusion Chemotherapy to Systemic Chemotherapy in Patients With Non Resectable Liver-only Colorectal Metastases](https://clinicaltrials.gov/ct2/show/NCT05103020) | NCT05103020 | Cetuximab | Recruiting | II | 1L+ | China | No | No | Yonsei University | Liver Met Positive | Conversion (Resectability) and Chemotherapy | Medical Device, FOLFIRI | Hepatic Arterial Infusion Pump, Topoisomerase, Antimetabolite | No | No | No | 11-01-21 | 03-01-25 |
| [Consolidative Radiotherapy for Colorectal Cancer Liver Metastases Receiving Surgery or Radiofrequency Ablation](https://clinicaltrials.gov/ct2/show/NCT03135652) | NCT03135652 | Cetuximab | Recruiting | II | Adjuvant/Neoadjuvant | China | No | No | Wuhan Union Hospital | Liver Met Positive | Chemotherapy and Radiotherapy | FOLFOX, CAPEOX, FOLFIRI, SBRT | Topoisomerase, Antimetabolite, DNA Alkylation | No | No | No | 08-01-17 | 06-01-23 |
| [Maintenance Therapy With 5-FU/FA Plus Panitumumab vs. 5-FU/FA Alone After Prior Induction and Re-induction After Progress for 1st-line Treatment of Metastatic Colorectal Cancer (PanaMa)](https://clinicaltrials.gov/ct2/show/NCT01991873) | NCT01991873 | Panitumumab | Active | II | 2L+ | Germany | Yes | Yes (Amgen) | AIO-Studien-gGmbH | All Comers | Rechallenge, Maintenance, and Chemotherapy | FOLFOX | Antimetabolite, DNA Alkylation | No | No | No | 04-01-14 | 12-31-22 |
| [Systemic Oxaliplatin or Intra-arterial Chemotherapy Combined With LV5FU2 +/- Irinotecan and an Target Therapy in First Line Treatment of Metastatic Colorectal Cancer Restricted to the Liver (OSCAR)](https://clinicaltrials.gov/ct2/show/NCT02885753) | NCT02885753 | Panitumumab | Recruiting | III | 1L | France | No | No | Federation Francophone de Cancerologie Digestive | All Comers | Chemotherapy | Medical Device, FOLFOX, FOLFOXIRI | Hepatic Arterial Infusion Pump, Topoisomerase, Antimetabolite, DNA Alkylation | No | No | No | 12-01-16 | 09-01-25 |
| [A Phase 1b, Open-Label, Dose-Escalation, Dose-Expansion Study Evaluating the Safety, Pharmacokinetics, and Efficacy of Orally Administered SM08502 Combined With Hormonal Therapy or Chemotherapy in Subjects With Advanced Solid Tumors](https://clinicaltrials.gov/ct2/show/NCT05084859) | NCT05084859 | Panitumumab | Recruiting | I | 2L+ | International | Yes | No | Biosplice Therapeutics, Inc. | All Comers | Targeted Agent and Chemotherapy | SM08502, FOLFIRI | CLK, Topoisomerase, Antimetabolite | No | No | No | 11-03-21 | 07-01-26 |
| [Panitumumab With or Without Trametinib in Treating Patients With Stage IV Colorectal Cancer](https://clinicaltrials.gov/ct2/show/NCT03087071) | NCT03087071 | Panitumumab | Recruiting | II | 2L+ | US | No | No | MD Anderson | All Comers | Targeted Agent | Trametinib | MEK1/2 | No | Yes | No | 12-29-17 | 07-01-24 |
| [FOLFIRI3 plus panitumumab or FOLFIRI3 plus bevacizumab as consolidation chemotherapy after preoperative chemoradiotherapy in patients with locally advanced rectal cancer; a phase II trial](https://citeline.informa.com/trials/details/363278?qId=0d4a15c7-852a-49d4-a38e-82c7e1fc9121) | N/A | Panitumumab | Open | II | Adjuvant/Neoadjuvant | Japan | No | No | Hospital/Medical Center | All Comers | Chemotherapy | FOLFIRI | Topoisomerase, Antimetabolite | No | No | No | 04-20-19 | 09-20-28 |
| [Panitumumab Skin Toxicity Prevention Trial (PaSTo)](https://clinicaltrials.gov/ct2/show/NCT03167268) | NCT03167268 | Panitumumab | Active | II | 1L+ | Italy | No | No | Ospedale San Carlo Borromeo | All Comers | Targeted Agent and Side Effect Management | Lycopene | Skin (Variable) | No | No | No | 08-03-16 | 11-30-21 |
| [Phase III Study to Evaluate FOLFOX + Panitumumab Followed by FOLFIRI + Bevacizumab Versus FOLFOX + Bevacizumab Followed by FOLFIRI + Panitumumab in Untreated Patients With Wild-type RAS Metastatic, Primary Left-sided, Unresectable Colorectal Cancer: The CR-SEQUENCE](https://clinicaltrials.gov/ct2/show/NCT03635021) | NCT03635021 | Panitumumab | Recruiting | III | 1L | Spain | Yes | Yes (Amgen) | Spanish Cooperative Group for the Treatment of Digestive Tumors | RAS/BRAF WT | Sequencing and Chemotherapy | FOLFOX, FOLFIRI | Topoisomerase, Antimetabolite, DNA Alkylation | No | No | No | 10-15-18 | 06-15-25 |
| [Monarcc: A Randomised Phase II Study of Panitumumab Monotherapy and Panitumumab Plus 5 Fluorouracil As First Line Therapy for RAS and BRAF Wild Type Metastatic Colorectal Cancer.](https://citeline.informa.com/trials/details/320789?qId=1fac9d9a-4bd4-4f82-8e40-7659c147547f) | N/A | Panitumumab | Open | II | 1L | New Zealand/Australia | Yes | Yes (Amgen) | Amgen | RAS/BRAF WT | Chemotherapy | 5FU | Antimetabolite | No | No | No | 07-16-18 | 09-01-22 |
| [Novel PET/CT Imaging Biomarkers of CB-839 in Combination With Panitumumab and Irinotecan in Patients With Metastatic and Refractory RAS Wildtype Colorectal Cancer](https://www.clinicaltrials.gov/ct2/show/NCT03263429) | NCT03263429 | Panitumumab | Recruiting | I/II | 2L+ | US | Yes | No | Vanderbilt-Ingram Cancer Center | RAS/BRAF WT | Targeted Agent | CB-839 | Glutaminase | No | No | No | 08-23-17 | 12-01-23 |
| [A Multicenter, Phase II Trial to Investigate the Safety and Efficacy of Panitumumab and Irinotecan in Neo RAS Wild Type Metastatic Colorectal Cancer Patients (C-PROWESS Study)](https://citeline.informa.com/trials/details/426245?qId=0d4a15c7-852a-49d4-a38e-82c7e1fc9121) | N/A | Panitumumab | Open | II | 2L+ | Japan | No | No | Hospital/Medical Center | RAS/BRAF WT | Chemotherapy | Irinotecan | Topoisomerase | No | No | No | 01-20-22 | 01-31-24 |
| [Trifluridine/ Tipiracil Plus Panitumumab Versus Trifluridine/ Tipiracil Plus Bevacizumab as First-line Treatment of Metastatic Colorectal Cancer](https://clinicaltrials.gov/ct2/show/NCT05007132) | NCT05007132 | Panitumumab | Recruiting | II | 1L | Germany | No | No | Dominik Paul Modest | RAS/BRAF WT | Chemotherapy | Trifluridin Tipiracil | DNA Synthesis | No | No | No | 12-17-21 | 12-31-32 |
| [FOLFOXIRI Plus Panitumumab in Metastatic RAS Wild-type, Left-sided Colorectal Cancer](https://clinicaltrials.gov/ct2/show/NCT04169347) | NCT04169347 | Panitumumab | Recruiting | II | 1L | US | Yes | Yes (Amgen) | Criterium, Inc. | RAS/BRAF WT | Chemotherapy | FOLFOXIRI | Topoisomerase, Antimetabolite, DNA Alkylation | No | No | No | 12-02-19 | 12-31-22 |
| [Panitumumab, Regorafenib, or TAS-102, in Treating Patients With Metastatic and/or Unresectable RAS Wild-Type Colorectal Cancer](https://clinicaltrials.gov/ct2/show/NCT03992456) | NCT03992456 | Panitumumab | Active | II | 2L+ | US | No | No | ACCRU | RAS/BRAF WT | Rechallenge | N/A | N/A | No | No | No | 04-24-20 | 10-07-23 |
| [Niraparib and Panitumumab in Patients With Advanced or Metastatic Colorectal Cancer (NIPAVect)](https://clinicaltrials.gov/ct2/show/NCT03983993) | NCT03983993 | Panitumumab | Recruiting | II | 2L+ | US | Yes | No | Emory University | RAS/BRAF WT | Targeted Agent | Niraparib | PARP | No | No | No | 10-15-19 | 10-31-24 |
| [Re-challenge Therapy With Chemotherapy & Panitumumab in Metastatic Colorectal Cancer Patients Treated With an Anti-EGFR (REPAN)](https://clinicaltrials.gov/ct2/show/NCT03940131) | NCT03940131 | Panitumumab | Recruiting | II | 3L+ | Saudi Arabia | No | No | King Abdullah Medical City | RAS/BRAF WT | Rechallenge and Chemotherapy | FOLFOX, FOLFIRI | Topoisomerase, Antimetabolite, DNA Alkylation | No | No | No | 06-10-20 | 07-01-22 |
| [FOLFOX + Panitumumab According to a "Stop and go" Strategy With a Reintroduction Loop After Progression on Fluoropyrimidine as Maintenance Treatment, as the First Line in Patients With Metastatic Colorectal Adenocarcinoma Without a RAS Mutation (OPTIPRIME)](https://clinicaltrials.gov/ct2/show/NCT03584711) | NCT03584711 | Panitumumab | Recruiting | II | 1L | France | Yes | Yes (Amgen) | Federation Francophone de Cancerologie Digestive | RAS/BRAF WT | Maintenance, Rechallenge, and Chemotherapy | FOLFOX | Antimetabolite, DNA Alkylation | No | No | No | 04-26-18 | 11-01-24 |
| [Study of medical treatment reactivity by the chemokine receptor (CXCR4) as first line treatment in patients with metastatic colorectal cancer](https://citeline.informa.com/trials/details/172061?qId=0d4a15c7-852a-49d4-a38e-82c7e1fc9121) | N/A | Panitumumab | Open | II | 1L | Japan | No | No | Academic Center | RAS/BRAF WT | Biomarker Selection and Chemotherapy | CXCR4+, FOLFOX | Antimetabolite, DNA Alkylation | No | No | No | 07-28-12 | N/A |
| [A Phase 2 Study of Panitumumab in Patients With Cetuximab-refractory Metastatic Colorectal Cancer (PACER)](https://clinicaltrials.gov/ct2/show/NCT01801904) | NCT01801904 | Panitumumab | Active | II | 2L+ | Italy | No | No | NCI (Naples) | RAS/BRAF WT | Sequencing | N/A | N/A | No | No | No | 12-01-12 | 12-01-22 |
| [First Line mFOLFOXIRI + PANITUMUMAB vs mFOLFOX + PANITUMUMAB IN RAS AND BRAF WT METASTATIC COLORECTAL CANCER PATIENTS (TRIPLETE)](https://clinicaltrials.gov/ct2/show/NCT03231722) | NCT03231722 | Panitumumab | Active | III | 1L | Italy | No | No | Gruppo Oncologico del Nord-Ovest | RAS/BRAF WT | Chemotherapy | FOLFOX, FOLFOXIRI | Topoisomerase, Antimetabolite, DNA Alkylation | No | No | No | 09-13-17 | 09-15-22 |
| [Second-line FOLFIRI + Panitumumab in Subjects With Wild Type RAS Metastatic Colorectal (BEYOND)](https://clinicaltrials.gov/ct2/show/NCT03751176) | NCT03751176 | Panitumumab | Active | II | 2L | Spain | Yes | Yes (Amgen) | Grupo Espanol Multidisciplinario del Cancer Digestivo | RAS/BRAF WT | Rechallenge and Chemotherapy | FOLFIRI | Topoisomerase, Antimetabolite | No | No | No | 11-08-18 | 11-30-22 |
| [FOLFOXIRI With or Without Panitumumab in Metastatic Colorectal Cancer (VOLFI) (VOLFI)](https://clinicaltrials.gov/ct2/show/NCT01328171) | NCT01328171 | Panitumumab | Active | II | 1L | Germany | Yes | Yes (Amgen) | AIO-Studien-gGmbH | RAS/BRAF WT | Chemotherapy | FOLFOXIRI | Topoisomerase, Antimetabolite, DNA Alkylation | No | No | No | 04-01-11 | 01-01-22 |
| [PAnitumumab REchallenge Followed by REgorafenib Versus the Reverse Sequence (PARERE)](https://clinicaltrials.gov/ct2/show/NCT04787341) | NCT04787341 | Panitumumab | Recruiting | II | 2L+ | Italy | No | No | Gruppo Oncologico del Nord-Ovest | RAS/BRAF WT | Sequencing | N/A | N/A | No | No | No | 12-15-20 | 06-15-24 |
| [Comparison FOLFIRINOX Panitumumab vs mFOLFOX6 Panitumumab in RAS/B-RAF Wild-type Metastatic Colorectal Cancer Patients (PANIRINOX)](https://clinicaltrials.gov/ct2/show/NCT02980510) | NCT02980510 | Panitumumab | Recruiting | II | 1L | France | No | No | UNICANCER | RAS/BRAF WT | Chemotherapy and ctDNA | FOLFIRINOX | Topoisomerase, Antimetabolite, DNA Alkylation | No | No | No | 12-01-16 | 01-01-24 |
| [Treatment Strategies in Colorectal Cancer Patients With Initially Unresectable Liver-only Metastases (CAIRO5)](https://clinicaltrials.gov/ct2/show/NCT02162563) | NCT02162563 | Panitumumab | Recruiting | III | 1L | Netherlands | No | No | Dutch Colorectal Cancer Group | RAS/BRAF WT and Liver Met Positive | Conversion (Resectability) and Chemotherapy | FOLFOX, FOLFIRI | Topoisomerase, Antimetabolite, DNA Alkylation | No | No | No | 07-01-14 | 07-01-25 |
| [Study of Hepatic Arterial Infusion With Intravenous Irinotecan, 5FU and Leucovorin With or Without Panitumumab, in Patients With Wild Type RAS Who Have Resected Hepatic Metastases From Colorectal Cancer](https://clinicaltrials.gov/ct2/show/NCT01312857) | NCT01312857 | Panitumumab | Active | II | 1L+ | US | Yes | Yes (Amgen) | MSKCC | RAS/BRAF WT and Liver Met Positive | Chemotherapy | Medical Device, FOLFIRI | Hepatic Arterial Infusion Pump, Topoisomerase, Antimetabolite | No | No | No | 03-01-11 | 03-01-23 |
| [Sotorasib and Panitumumab Versus Investigator's Choice for Participants With Kirsten Rat Sarcoma (KRAS) p.G12C Mutation (CodeBreak 300)](https://clinicaltrials.gov/ct2/show/NCT05198934) | NCT05198934 | Panitumumab | Recruiting | III | 2L+ | International | Yes | Yes (Amgen) | Amgen | RAS Mut | Targeted Agent | Sotorasib | KRAS G12C | No | Yes | No | 04-19-22 | 03-28-24 |
| [FIRE-5 -Study: Optimal Anti-EGFR Treatment of mCRC Patients With Low-Frequency RAS Mutation](https://clinicaltrials.gov/ct2/show/NCT04034173) | NCT04034173 | Panitumumab | Not Yet Recruiting | II | 1L | Germany | Yes | Yes (Amgen) | Univeristy of Munich | RAS Mut | Biomarker Selection and Chemotherapy | FOLFIRI | Topoisomerase, Antimetabolite | No | No | No | 08-01-19 | 08-01-26 |
| [A Multicenter Phase I/II Study of Cabozantinib Alone or in Combination with Panitumumab in Patients with MET-amplified Metastatic Colorectal Cancer](https://citeline.informa.com/trials/details/346631?qId=1fac9d9a-4bd4-4f82-8e40-7659c147547f) | N/A | Panitumumab | Open | I/II | 1L+ | Japan | No | No | National Cancer Center Hospital East | MET Amplified | Targeted Agent | Cabozantinib | Multikinase | No | No | No | 04-22-19 | 01-31-23 |
| [A Safety, Pharmacokinetic and Efficacy Study of NUC-3373 in Combination With Standard Agents Used in Colorectal Cancer Treatment](https://clinicaltrials.gov/ct2/show/NCT03428958) | NCT03428958 | Both | Recruiting | I/II | 2L+ | International | Yes | No | NuCana plc | All Comers | Chemotherapy | NUC-3373 with oxaliplatin or irinotecan | Topoisomerase, Antimetabolite, DNA Alkylation | No | No | No | 10-16-18 | 12-01-22 |
| [Rational Anti-EGFR Therapy Selection for the First-line Treatment of Patients with Metastatic KRAS/NRAS Wild Type Colorectal Cancer Based on the Use of Molecular Predictor miR-31-5p (RASmiR)](https://citeline.informa.com/trials/details/353771?qId=0d4a15c7-852a-49d4-a38e-82c7e1fc9121) | N/A | Both | Open | II | 1L | Europe | No | No | Hospital/Medical Center | RAS/BRAF WT | Biomarker Selection | miR-31-5p | N/A | No | No | No | 06-30-19 | N/A |
| [Multi-Line Therapy Trial in Unresectable Metastatic Colorectal Cancer (STRATEGIC-1)](https://clinicaltrials.gov/ct2/show/NCT01910610) | NCT01910610 | Both | Recruiting | III | 1L | Europe | Yes | No | GERCOR - Multidisciplinary Oncology Cooperative Group | RAS/BRAF WT | Chemotherapy and Sequencing | FOLFIRI, Irinotecan | Topoisomerase, Antimetabolite | No | No | No | 10-30-13 | 06-01-21 |
| [Regorafenib, With Cetuximab or Panitumumab, for the Treatment of Unresectable, Locally Advanced, or Metastatic Colorectal Cancer](https://clinicaltrials.gov/ct2/show/NCT04117945) | NCT04117945 | Both | Recruiting | II | 2L+ | US | No | No | ACCRU | RAS/BRAF WT | Chemotherapy and Sequencing | Irinotecan | Topoisomerase | No | No | No | 03-03-20 | 03-31-25 |
| [Early-Line Anti-EGFR Therapy to Facilitate Retreatment for Select Patients With mCRC](https://clinicaltrials.gov/ct2/show/NCT04587128) | NCT04587128 | Both | Recruiting | II | 1L+ | US | No | No | University of Wisconsin, Madison | RAS/BRAF WT | Chemotherapy and Sequencing | Irinotecan | Topoisomerase | No | No | No | 10-19-20 | 10-01-25 |
| [A Prospective Study Utilizing Circulating Cell Free DNA (cfDNA) Use in the Detection of RAS Mutations in Patients With Advanced Colorectal Cancer.](https://clinicaltrials.gov/ct2/show/NCT04775862) | NCT04775862 | Both | Recruiting | II | 2L+ | Saudi Arabia | No | No | National Guard Health Affairs | RAS/BRAF WT | ctDNA and Sequencing | N/A | N/A | No | No | No | 02-21-21 | 02-01-24 |
| [Randomised Phase II Trial to Evaluate Progression-Free Survival in Integrating Local Ablative Therapy with First-Line Systemic Treatment for Unresectable Oligometastatic Colorectal Cancer](https://citeline.informa.com/trials/details/414521?qId=1fac9d9a-4bd4-4f82-8e40-7659c147547f) | N/A | Both | Open | II | 1L | New Zealand/Australia | No | No | Australasian Gastrointestinal Trials Group | RAS/BRAF WT and Metastasis Positive | Local Ablative Therapy and Chemotherapy | Local Ablative Therapy, FOLFOX, FOLFIRI | Radiation, Topoisomerase, Antimetabolite, DNA Alkylation | No | No | No | 11-01-21 | 11-01-26 |
| [A Study of the Use of the Medtronic Pump and Codman Catheter to Give Chemotherapy to Patients With Colorectal Carcinoma or Cholangiocarcinoma](https://clinicaltrials.gov/ct2/show/NCT04668976) | NCT04668976 | Both | Recruiting | II | 1L+ | US | No | No | Benaroya | RAS/BRAF WT and non-MSI-H/dMMR | Chemotherapy | Medical Device, FOLFIRI, FOLFOX, irinotecan, oxaliplatin | Hepatic Arterial Infusion Pump, Topoisomerase, Antimetabolite, DNA Alkylation | No | No | No | 11-25-20 | 12-01-24 |
| [A Study of the Use of the Medtronic Pump and Codman Catheter to Give Chemotherapy to Patients With Colorectal Carcinoma or Cholangiocarcinoma](https://clinicaltrials.gov/ct2/show/NCT03693807) | NCT03693807 | Both | Active | II | 1L+ | US | No | No | MSKCC | RAS/BRAF WT and non-MSI-H/dMMR | Chemotherapy | Medical Device, FOLFIRI, FOLFOX, irinotecan, oxaliplatin | Hepatic Arterial Infusion Pump, Topoisomerase, Antimetabolite, DNA Alkylation | No | No | No | 10-18-18 | 09-01-22 |
| [Hepatic Artery Infusion Pump Chemotherapy With Floxuridine and Dexamethasone in Combination With Systemic Chemotherapy for Patients With Colorectal Cancer Metastatic to the Liver](https://clinicaltrials.gov/ct2/show/NCT03366155) | NCT03366155 | Both | Recruiting | II | 2L+ | US | No | No | NCI | Non-MSI-H/d-MMR | Chemotherapy | Medical Device, floxuridine, dexamethasone | Hepatic Arterial Infusion Pump, Antimetabolite, Steroid | No | No | No | 06-24-19 | 12-30-25 |
