# Supplementary material for: Anti-EGFR Antibodies in the Management of Advanced Colorectal Cancer
Source: Oncologist. 2023 Sep 29;28(12):1034–48. doi: 10.1093/oncolo/oyad262 (PMC11025386; doi:10.1093/oncolo/oyad262)
Supplement: oyad262_suppl_Supplementary_Material [file oyad262_suppl_supplementary_material.docx]

**Supplementary material**

The keywords used in the Trialtrove were ‘oncology’ therapeutic area and ‘colorectal’ disease state. Trials status of ‘open’ or ‘planned’ was included for phase I, I/II, II, II/III, and III. The tested drug was either ‘cetuximab’ or ‘panitumumab’. The clinicaltrials.gov search was for any trial containing the drug ‘cetuximab’ or ‘panitumumab’. These search databases were cross referenced to remove duplicate trials. A spreadsheet (**Supplemental Table 1**) was generated and the NCT identifier, EGFR-targeted compound, trial status, phase, line of therapy, location, trial sponsor, CRC disease population, trial type, combination partner, therapeutic target, and trial start/end date were recorded. Trials where cetuximab or panitumumab were only in the control arm were excluded. Further, trials discussing development of biosimilars were not included in this analysis. These were quantified and visualized in GraphPad Prism.

A detailed report listing all the included clinical trials is added in the **Supplementary Table 1** below.
